# Severe Fertility Effects of *sheepish* Sperm Caused by Failure To Enter Female Sperm Storage Organs in *Drosophila melanogaster*

**DOI:** 10.1534/g3.117.300171

**Published:** 2017-11-20

**Authors:** Masatoshi Tomaru, Takashi Ohsako, Masahide Watanabe, Naoto Juni, Hiroshi Matsubayashi, Hiromi Sato, Ayako Takahashi, Masa-Toshi Yamamoto

**Affiliations:** Department of Drosophila Genomics and Genetic Resources, Center for Advanced Insect Research Promotion, Kyoto Institute of Technology, 616-8354, Japan

**Keywords:** *Drosophila*, male sterility, sperm storage, sperm motion, flavin-containing amine oxidase

## Abstract

In *Drosophila*, mature sperm are transferred from males to females during copulation, stored in the sperm storage organs of females, and then utilized for fertilization. Here, we report a gene named *sheepish* (*shps*) of *Drosophila melanogaster* that is essential for sperm storage in females. *shps* mutant males, although producing morphologically normal and motile sperm that are effectively transferred to females, produce very few offspring. Direct counts of sperm indicated that the primary defect was correlated to failure of *shps* sperm to migrate into the female sperm storage organs. Increased sperm motion parameters were seen in the control after transfer to females, whereas sperm from *shps* males have characteristics of the motion parameters different from the control. The few sperm that occasionally entered the female sperm storage organs showed no obvious defects in fertilization and early embryo development. The female postmating responses after copulation with *shps* males appeared normal, at least with respect to conformational changes of uterus, mating plug formation, and female remating rates. The *shps* gene encodes a protein with homology to amine oxidases, including as observed in mammals, with a transmembrane region at the C-terminal end. The *shps* mutation was characterized by a nonsense replacement in the third exon of *CG13611*, and *shps* was rescued by transformants of the wild-type copy of *CG13611*. Thus, *shps* may define a new class of gene responsible for sperm storage.

Sperm storage is an essential process in the reproduction of *Drosophila*. During copulation, a male transfers sperm to the female, where they are stored in the sperm storage organs. In *Drosophila melanogaster*, females have two types of sperm storage organ, a single seminal receptacle on the ventral side, a blind-ended tube of ∼2 mm-long, and two spermathecae of mushroom-shaped, cuticular, dorsal-side organs surrounded by secretory cells, both of which are connected to the uterus (Miller 1950; Kiefer 1969; Neubaum and Wolfner 1998; Bloch Qazi *et al.* 2003; Wolfner 2009; Schnakenberg *et al.* 2012). During copulation, 1500–4000 sperm are transferred from the male and one-quarter to one-third of them are stored in the seminal receptacle and the pair of spermathecae (Lefevre and Jonsson 1962; Kiefer 1969; Neubaum and Wolfner 1998; Bloch Qazi *et al.* 2003; Wolfner 2009; Schnakenberg *et al.* 2012). Sperm remaining in the uterus are ejected before oviposition (Manier *et al.* 2010), and, therefore, movement into the storage organs is a critical factor determining female fertility. During ovulation, only a few sperm are discharged from the sperm storage organs (Lefevre and Jonsson 1962; Kiefer 1969; Bloch Qazi and Wolfner 2003; Ohsako and Yamamoto 2011). Not only the head but also the whole body of the sperm (ca. 2 mm long, Kiefer 1969; Joly *et al.* 2004), including its plasma membrane, enters the egg (Perotti 1975). There, it persists as a coiled structure for some time in the cytoplasm of the inseminated egg (Karr 1991; Graner *et al.* 1994; Ohsako *et al.* 2003; Ohsako and Yamamoto 2011).

Mature sperm are stored in the seminal vesicle, the sperm storage organ of males, where motile sperm can be observed (Lefevre and Jonsson 1962), and it is well known that sperm motility is a critical factor essential for sperm storage in the female (Neubaum and Wolfner 1998; Bloch Qazi *et al.* 2003; Wolfner 2009; Schnakenberg *et al.* 2012). In mammals, hyperactivation and capacitation of sperm have been extensively studied, and are known to play a pivotal role in fertilization. Regulation of activation and capacitation in mammalian systems involves separate Ca${}^{2+}$ pathways (Marquez and Suarez 2004), and is dependent on internal Ca${}^{2+}$ concentration and reactive oxygen species (Aitken 2000; Publicover *et al.* 2007; Aitken *et al.* 2012). A similar process of activation in *D. melanogaster*, which also exhibits increased flagellar beat frequency after transfer to the female, has been suggested by Köttgen *et al.* (2011). However, our understanding of these processes in *Drosophila* remains poor. Both sexes affect the process of sperm storage in several steps (Neubaum and Wolfner 1998; Bloch Qazi *et al.* 2003; Wolfner 2009; Schnakenberg *et al.* 2012). In females, sperm storage is affected by substances from spermathecae, spermathecal duct, and parovaria (Allen and Spradling 2008; Iida and Cavener 2004; Schnakenberg *et al.* 2011; Sun and Spradling 2013). The nervous system of females is also required for normal sperm storage (Arthur *et al.* 1998; Rodríguez-Valentín *et al.* 2006; Avila *et al.* 2012). In males, seminal fluid proteins secreted by male accessory glands and the ejaculatory duct are important for postmating responses, such as sperm entry to the female, sperm storage, maintenance and release of sperm, sperm usage, ovulation, and behavioral changes in females (Aigaki *et al.* 1991; Neubaum and Wolfner 1998; Bloch Qazi *et al.* 2003; Ravi Ram and Wolfner 2009; Wolfner 2009; Schnakenberg *et al.* 2012). For example, Esterase 6 (Gilbert 1981) and Acp36DE (Neubaum and Wolfner 1999; Bloch Qazi and Wolfner 2003), and other accessory gland proteins (ACPs), are important for sperm storage, and Acp29AB is important for the maintenance of sperm storage, but not for sperm entry into sperm storage organs (Wong *et al.* 2008).

We previously reported two mutations categorized as postspermatogenesis male-sterile mutants, *misfire* (*mfr*), and *wasted* (*wst*), which were screened from EMS-induced and natural populations, respectively (Ohsako *et al.* 2003; Ohsako and Yamamoto 2011). *mfr* is a paternal effect mutation that prevents formation of a male pronucleus after entry into an egg (Ohsako *et al.* 2003), while *wst* sperm enter the female sperm storage organs but are rapidly discharged from there (Ohsako and Yamamoto 2011).

Here, we report a gene named *sheepish* (*shps*), known as *CG13611* in FlyBase (Gramates *et al.* 2017), that is essential for sperm storage in females. *shps* sperm are motile after transfer to the uterus but rarely enter the storage organs of the female, resulting in male sterility. However, sperm that occasionally enter the female sperm storage organs are fertilization competent, suggesting that *shps* is related to processes required for sperm entry and storage. The phenotype of *shps* resembles that of *male fertility factor kl1* (*kl-1*, Kiefer 1969) that are suggested to be *WD40 Y* (*WDY*), which encodes WD40-rich proteins (Vibranovski *et al.* 2008), *Polycystic kidney disease 2 ortholog* (*Homo sapiens*) (*Pkd2*), a homolog of the human polycystic kidney disease 2 gene (PKD2) that encodes a cation (calcium) channel protein, TRPP2, a member of the transient receptor potential (TRP) family (Gao *et al.* 2003; Watnick *et al.* 2003; Köttgen *et al.* 2011; Yang and Lu 2011), and *lost boy* (*lobo*), an ortholog of vertebrate Ccdc135, or *Chlamydomonas reinhardtii* FAP50, which encodes a protein associated with outer doublet microtubules of flagellum (Yang *et al.* 2011). Although similar in phenotype, sequence homology suggests *shps* encodes a flavin-containing amine oxidase with a C-terminal transmembrane region, and is unrelated to the aforementioned proteins. Thus, *shps* identifies a new functional category of protein essential for fertility and sperm storage in the *Drosophila* female.

## Materials and Methods

### Drosophila stocks

All cultures were raised at room temperature (between 23 and 25°) on a standard corn-glucose-yeast medium. The *shps* mutation was isolated in a screen designed to recover ethyl methanesulfonate-induced male sterile mutations (*cf*. Ohsako *et al.* 2003; Hirai *et al.* 2004; Ohsako and Yamamoto 2011). We used three strains, but an identical allele of *shps*: (i) *shps/TM3*, *Sb Ser*, (ii) *y*^∗^ *w*^∗^*; P{protamineB-eGFP}; shps/TM3, Sb Ser*, (iii) *C(1)RM*, *y v f/C(1*;*Y)6*; *shps/TM3*, *Sb Ser*. Crosses between *shps/TM3* or *shps* females and *y*^∗^ *w*^∗^*; protamineB-eGFP; shps/TM3* males were made to obtain flies with *protamineB-eGFP*. The Oregon-R stock was used as a wild-type control and females for crosses, unless otherwise mentioned. A third chromosome multiple marker strain, *ru h th st cu sr* $e^{s}$ *ca/TM3, Sb Ser*, was used for recombination mapping. Eight deficiencies used to map the mutation, and their breakpoints were as follows: *In(3R)Ubx^7LL^* ats*^R^* (96A1;96A25 for deleted segment and 89C;89E2 for duplicated segment), *Df(3R)crb87-5* (95F5;96A18), *Df(3R)Exel8178* (95F8;96A6), *Df(3R)Exel6199* (95F8;96A2), *Df(3R)crb-F89-4* (95D7;95F15), *Df(3R)crb87-4* (95D11–E2;96A2), *Df(3R)Exel6198* (95E5;95F8), and *Df*(*3R*)*P3-4-7*, *P{lacW}degenerated* (95F6-7;95F10, see Supplemental Material, File S1). The left ends of *Df(3R)Exel8178* and *Df(3R)Exel6199* and the right end of *Df(3R)Exel6198* are identical. The insertion point of the starter insertion, *P{XP}jar^d02406^*, was used to generate these deficiencies (Parks *et al.* 2004). Two male sterile or lethal mutation strains, *ry^506^ P{PZ}jar^1^/TM3, ry^RK^ Sb Ser* (95F6-8) and *y w^1118^; P{lacW}crb^j1B5^/TM3, Sb* (95F11-12), were used for a complementation test. A *GFP-myosin VI* (*P{Hsp83-jar.GFP}*) line, kindly provided by K. Miller at Washington University, was used to test whether *GFP-myosin VI* rescues the sterility of *shps*. Descriptions of the genes and chromosome rearrangements are available in FlyBase (http://flybase.org/, Gramates *et al.* 2017).

To examine sterility, the females that copulated with the males of each genotype were singly placed in culture vials, and transferred to new vials at 3-d intervals. All adult flies emerging from the culture vials were counted. Copulated females were dissected, and their seminal receptacle and spermathecae were observed for sperm presence under a phase-contrast microscope.

### Hatchability and egg to adult rate

All the flies were used 3–5 d after emergence. The females that copulated at least for 15 min with the males of each genotype (wild-type, *shps/TM3* or *shps*) were placed singly in culture vials. They were allowed to lay eggs for 24 hr, and transferred to new vials every day. We counted the number of eggs laid, and the next day, the number of hatched eggs was counted. All adult flies emerging from the culture vials were counted.

### Quantification of stored sperm

**Figure 1:**
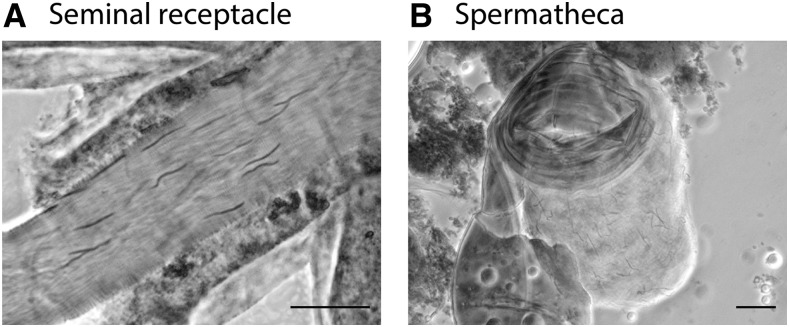
Lacto-acetic orcein staining of seminal receptacle (A) and spermatheca (B). Heads of sperm have a needle-like shape and are distinguishable from female somatic cells. Bar, 20 μm.

Females were dissected 1 hr or 24 hr after copulation with *shps/TM3* or *shps* males. Spermathecae and seminal receptacles were stained with lacto-acetic orcein, and then counted for heads of the sperm in the female sperm storage organs. Heads of sperm were distinguished from the nuclei of other tissues of the females by their needle-like shape (Figure 1).

### Observation of uterus morphology and mating plug

The uterus changes its morphology during mating, and Acp36DE is required for this morphological transition of the uterus (Adams and Wolfner 2007; Avila and Wolfner 2009). Three- to 5-d-old females that copulated with *protamineB-eGFP/+*; *shps/TM3* or *protamineB-eGFP/+*; *shps* males were frozen at –18°, 20 min after start of mating (ASM 20). The females were dissected, and the uterus and vagina were placed on a glass slide and covered with a drop of halocarbon oil 700 (Sigma). The female reproductive organs were observed under a phase contrast Nikon Eclipse 80i microscope.

A male ejaculatory bulb protein, PEB-me, is a major component of the mating plug, and shows autofluorescence (Lung and Wolfner 2001), which serves as a proxy for the presence of a mating plug. Female reproductive organs showing autofluorescence were also counted under epifluorescent optics on a Nikon Eclipse 80i microscope with a UV-1A filter (Ex 365/10, DM 400, and BA 400). After observation of the uterus morphology and mating plug, the number of sperm was counted as described below.

### Observation of sperm during copulation in the sperm storage organs of females

Females were allowed to copulate with *protamineB-eGFP/+*; *shps/TM3* or *protamineB-eGFP/+*; *shps* males. Copulating pairs in 15 min (ASM 15) or ASM 20 since copulation began, and females within 10 min of copulation end were frozen at –18°, and the females were then dissected for observation. To visualize fluorescence of *protamineB-eGFP* on sperm heads, the female reproductive organs were observed under epifluorescent optics on a Nikon Eclipse 80i microscope with an FITC filter (EX 465–495, DM 505, and BA 515–555). We counted the number of sperm in the uterus, seminal receptacle, and spermathecae. The number of sperm in seminal vesicle of unmated males was also counted.

### Quantification of sperm motion

**Figure 2:**
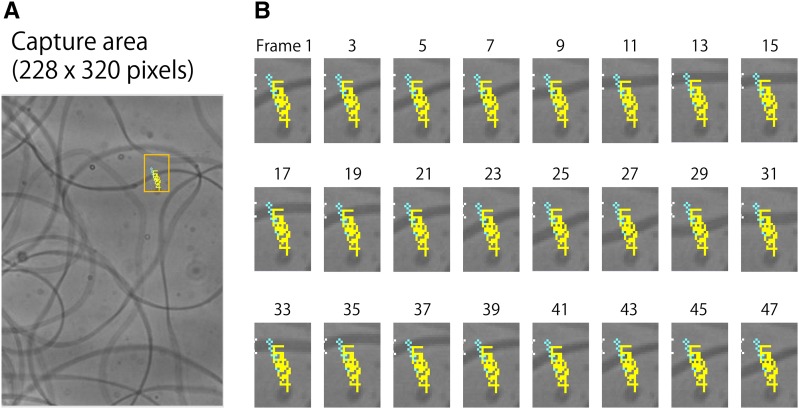
Sperm motion analysis. (A) A captured photograph was 228 × 320 pixels. Intensity index was measured with a Chinou Jouhou Shisutemu Motion Analyzer for a 228 × 320 pixel area, which is the same size as (A); 28 pixels correspond to 10 μm. (B) To quantify sperm beat frequency and beat speed, five measurement points were selected, and intensities of their pixels were measured using DITECT DippMotionPro 2D ver. 2.25. A high-speed camera captured 300 frames per second. The two frame interval of the continuous photographs shown here is 1/150 sec. Continuous photographs were trimmed only for the purpose of presentation. Intensity changes at measurement points could be detected as zero crossing frequency, that is, sperm beat frequency in Hertz. Beat speed was calculated using an actual distance (10 μm/28 pixels) and observation time (1000 frames = 1000/300 sec).

Motion of sperm from males of unmated 5-d-old males was measured following dissection from the seminal vesicle into Ringer’s solution on a glass slide. Motion of sperm following transfer to the females was similarly measured in single-pair matings within 15 min of completion of copulation. Sperm were dissected from the uteri of Canton-S females and placed in a droplet of Ringer’s solution on a slide glass. Photographs were captured continuously using a DITECT HAS-220 high-speed monochrome camera at 300 frames/s, 228 × 320 pixels for 1000 frames, using a Nikon Eclipse E800 microscope. The brightness changes were measured from the captured photographs using DITECT DippMotionPro 2D ver. 2.25 software. Motion parameters were measured by changes in pixel intensity responding to sperm motion in the field of view. Three parameters characterizing sperm motion were measured (Figure 2). (i) The motion of sperm tail in the area could be detected as zero crossing frequency, which was defined as sperm beat frequency in Hz. (ii) Actual distances were converted from the pixel size (28 pixels/10 μm) and tail beat speed at the point of focus was calculated in micrometer per second. (iii) Many sperm were observed in the area, and each showed motion differently. To estimate the intensity of sperm motion as a whole, all observed pixel brightness data were used for calculation using Motion Analyzer software (Chinou Jouhou Shisutemu, Japan), which was defined by the following formula:

$$\text{Intensity}\;\text{index}=\frac{1}{m\left(n-d\right)}\sum_{i=d+1}^{n}\sum_{j=1}^{m}\frac{\left|p_{j}^{i}-p_{j}^{i-d}\right|}{g}\times 100,$$

where *d* is the interval of the number of frames, *i* is the frame i.d. number, *n* is the number of frames analyzed (1000), *j* is the individual pixel i.d. number, *m* is the number of pixels analyzed per frame (72,960), *g* is the brightness depth (256), and $p_{j}^{i}$ is each brightness of frame i.d. *i* and pixel i.d. *j*.

### Egg collection and staining

Observation of sperm that entered an egg was carried out as per previous reports (Ohsako *et al.* 2003; Ohsako and Yamamoto 2011). Twenty copulated females (3–5 d old) were placed in culture vials for 1 hr. They were then transferred to Petri dishes containing fresh egg-laying medium (25% apple juice, 1.25% sucrose, 1.75% agar) with yeast paste. Eggs laid by females mated with males of each genotype were collected within 5 hr of egg deposition, and stored at 4° to prevent the embryos from developing further for up to 2 hr before fixation. Eggs were washed in 1 × PBS (1.9 mM NaH${}_{2}$PO${}_{4},$ 8.4 mM Na${}_{2}$HPO${}_{4},$ 175 mM NaCl, pH 7.4) containing 0.05% Tween-20 (PBST), and dechorinated in 50% commercial bleach for 2 min. Dechorinated eggs were washed and transferred into a two-phase fixative of heptane/PBS containing 3.7% formaldehyde and shaken for 20 min. The vitelline membranes were removed by transfer into a methanol/heptane mixture and vigorous shaking until most of the eggs sank to the bottom of the methanol layer. Eggs were washed three times with methanol and stored in methanol at 4° until use.

The eggs were stained with a mouse monoclonal antibody, DROP1.1, which recognizes the sperm tail in inseminated eggs (Karr 1991; Graner *et al.* 1994). Eggs were then incubated in DROP1.1 primary antibody for 1 hr, washed in PBST for 1 hr, and subsequently stained with Alexa Fluor 488 (Molecular Probe) for 30 min. The eggs were counter-stained with 1 μg/ml 4′,6-diamidine-2-phenylindole (DAPI) for 5 min to examine whether the inseminated eggs initiated mitotic division. The stained eggs were mounted under coverslips with 90% glycerol in PBS. All the staining and washing was performed at room temperature. Eggs were observed using epifluorescent optics on a Nikon Eclipse E600 microscope with filters for FITC (Ex 465–495, DM 505, and BA 515–555) and DAPI (Ex 340–380, DM 400, and BA 435–485).

### Remating tests using XO flies

The *shps/TM3* females were mated with *C(1*;*Y)6/O*; *shps/TM3* males, to produce lacking a *Y* chromosome: genotype, *X/O*; *shps/TM3* and *X/O*; *shps*. Since XO males do not produce sperm, but do produce seminal fluid, *X/O*; *shps* males were used in matings with wild-type females, and remating frequencies were measured and compared to similar matings using *X/O*; *shps/TM3* males. Three- to 5-d-old virgin females were mated with wild-type, *shps/TM3*, *shps*, *X/O*; *shps/TM3* or *X/O*; *shps* males. The next day (14–18 hr after mating), each female was introduced singly into an empty vial with a wild-type male. For each crossing, we observed 100 pairs and counted the number of mated pairs within 30 min. Virgin females of the same age as the females used for the remating test were used as a control of the second mating.

### DNA sequencing

Genomic DNA samples were isolated from homozygous *shps* males and regions of *CG13611* were PCR-amplified using KOD -Plus- DNA polymerase (Toyobo) with the following primer set: left primer: 5′-GCTATACGCTGCTCCTCTTCACTT-3′ and right primer: 5′-GAGCAGAGCGAACTTCTGAATGGA-3′. The amplified fragments were purified using QIAquick Gel Extraction Kit (Qiagen), and treated with Applied Biosystems BigDye Terminator v3.1. The nucleotide sequence was determined with an Applied Biosystems 3130xl Genetic Analyzer.

### Rescue experiment

For constructing the *pUAST-attB-loxP* vector, a 100-bp fragment containing the *attB* core sequence was amplified by PCR from the *attB-P[acman]-Cm^R^* vector (Venken *et al.* 2006) using the primers P1: 5′-ATTTCACACCGCATAATAACTTCGTATAGCATACATTATACGAAGTTATGTAGGTCACGGTCTCGAAGCCGCG-3′ and P2: 5′-TGAGAGTGCACCATAGTTCATCATGATGGACCAGATGGG-3′. Both the primers were added terminally, with 15-bp sequences homologous to the flanking sequences of the *Nde*I site of the *pUAST* vector, and the P1 primer was added internally, with a 34-bp *loxP* site. The amplified fragment was subcloned into the *Nde*I site of the *pUAST*, including a putative ORF as well as a 2.0-kb upstream and a 1.0-kb downstream region of *CG13611*, was amplified by PCR from Oregon-R genomic DNA using the primers shps-up: 5′-ATTCGTTAACAGATCGCGTTGGCTGCAGATTGCTATAAC-3′ and shps-dw: 5′-CTTGAGCTCGAGATCCCTTGCCACAATTGCTGCTCACTT-3′ (Figure S1A in File S1). Both the primers were added terminally with 15-bp sequences homologous to the flanking sequences of the *Bgl*II site of the *pUAST-attB-loxP* vector. The amplified fragment was cloned into the *Bgl*II site of the *pUAST-attB-loxP* vector using the In-Fusion HD Cloning Kit. The construct in *pUAST-attB-loxP* was injected into embryos of *y M{vas-int.Dm}ZH-2A w${}^{*}$; PBac{y${}^{+}$-attP-3B}VK00037*.

Females that copulated with males bearing the transgene containing the wild-type allele of the *CG13611* gene on the *shps* mutant background were placed individually into vials, and allowed to lay eggs for 5 d, then transferred to new vials for an additional 5 d. All of the flies that emerged from each vial were counted to examine male fertility. Two independently established transgenic lines were tested.

### Data availability

All strains are available on request (Table S2 in File S2). Accession numbers for sequences are as follows: DDBJ/GenBank/EMBL accession No. AB932860 (genome region of *CG13611*), AB932858 (5′ end of the P3-4-7 insertion) and AB932859 (3′ end of the P3-4-7 insertion). File S1 contains details of determination of the break point ends of deficiency, *Df(3R)P3-4-7*, *P{lacW}degenerated* and Figure S1. Figure S1 in File S1contains a schematic map of deficiencies used for mapping and a genome map, and detection of PCR amplification from P3-4-7 and *P{lacW}crb${}^{j1B5}$* flies. File S2 contains Figure S2, Figure S3, Table S1, and Table S2 and the legend of the movie (File S3). Figure S2 in File S2 contains protein structures of Shps and its homologous proteins of 12 species of *Drosophila* and human monoamine oxidases. Figure S3 in File S2 contains protein sequence alignment of Shps and its homologous proteins of 12 species of *Drosophila* and human monoamine oxidases. Table S1 in File S2 contains multiple comparisons by Dunn’s methods after Kruskal-Wallis test for sperm motion parameters. Table S2 in File S2 contains strains used in this study. File S3 contains a sperm motion video. Other data that support our findings are described in the *Results*.

## Results

### Characteristics of the shps phenotype

**Figure 3:**
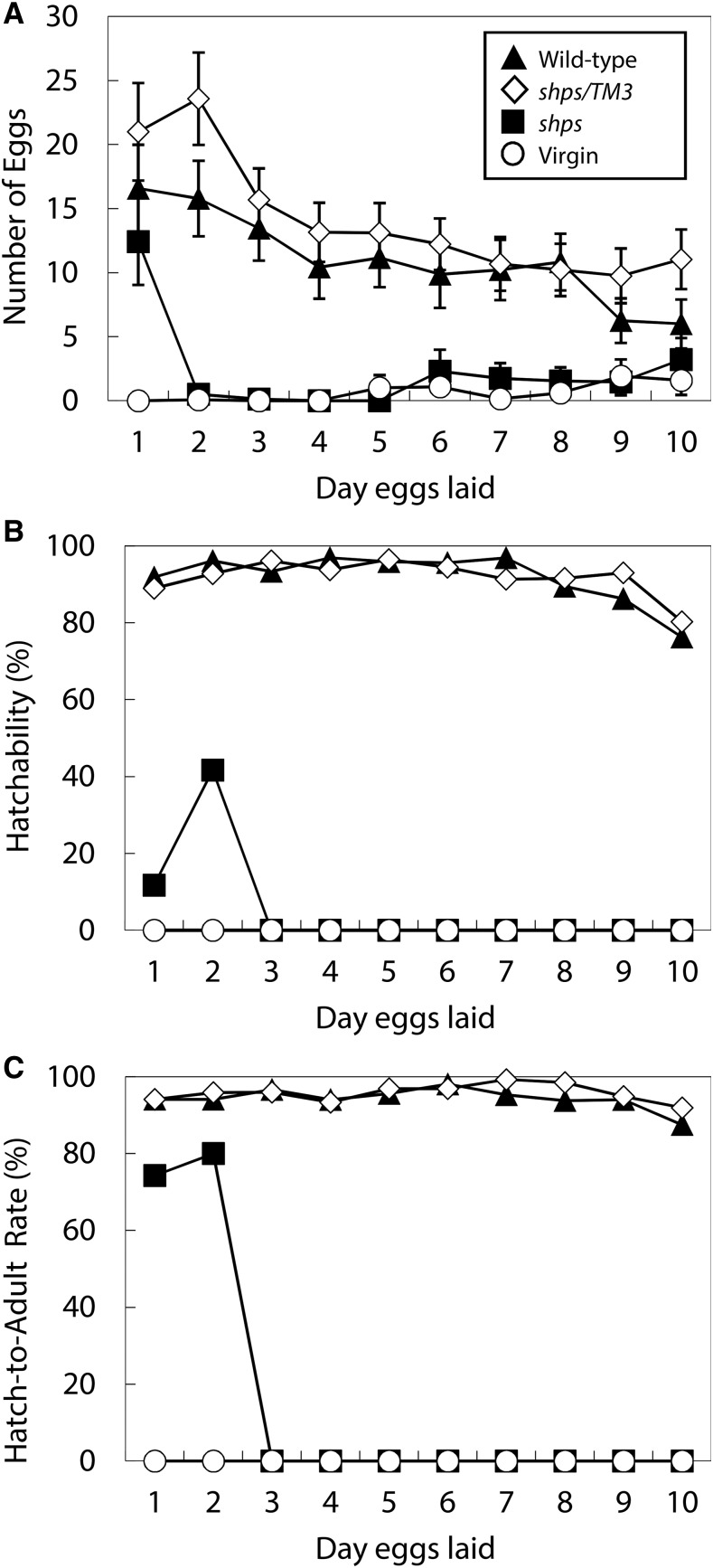
Oviposition, hatchability, and viability. (A) Number of eggs laid. Error bars are SE. (B) Hatchability (%). Number of hatched eggs was counted the day after the eggs were laid. (C) Hatch-to-adult rate (%). All emerged adult flies were counted. In the case there were no hatched eggs, hatch-to-adult rates are shown as 0% (*shps* for d 3–10 and virgin for all data). Filled triangle, eggs from the females that copulated with wild-type males (*N* = 28); open diamond, those with *shps/TM3* males (*N* = 28); filled box, those with *shps* males (*N* = 24); open circle, virgin females (*N* = 25).

Although *shps* was screened as a male sterile mutation, sterility is not 100% and homozygous males produce a small number of progeny (Table 1). To determine the stage of development disrupted by *shps*, the number of eggs laid, hatchability, and hatch-to-adult rate were evaluated (Figure 3). Wild-type females crossed with wild-type or *shps/TM3* males laid on average 110.5 ± 13.8 eggs (*N* = 28) and 140.4 ± 14.5 eggs (*N* = 28), respectively, over a 10-d period. In contrast, females mated with *shps* males laid on average 23.3 ± 6.2 eggs (*N* = 24), the majority laid on d 1 and a few on the following days. Virgin females laid a few eggs (6.4 ± 2.9, *N* = 25) in the last half of the observation (aged 9–15 d old).

**Table 1:** Number of progeny and sperm storage in the females

Cross*^a^*	Number of Progeny*^b^*	Sperm Storage*^c^*	Breakpoints*^d^*
Wild-type	186.2 ± 2.7 (14)	+++	–
*shps/TM3*	156.1 ± 4.4 (14)	+++	–
*shps*	1.5 ± 0.6 (12)	±	–
*protamineB-eGFP/+*; *shps/TM3*	123.0 ± 3.6 (12)	+++	–
*protamineB-eGFP/+*; *shps*	8.8 ± 0.6 (12)	±	–
*In(3R)Ubx${}^{7LL}$*ats${}^{R}$/shps	90.6 ± 27.5 (12)	+++	96A1;96A25
*Df(3R)crb-F89-4/shps*	3.0 ± 1.4 (12)	±	95D7;95F15
*Df(3R)crb87-4/shps*	2.8 ± 0.9 (12)	±	95D11-E2;96A2
*Df(3R)crb87-5/shps*	2.6 ± 1.1 (12)	±	95F7;96A18
*Df(3R)Exel6199/shps*	8.9 ± 3.6 (12)	±	95F8;96A2
*Df(3R)Exel8178/shps*	10.2 ± 2.9 (13)	±	95F8;96A6
*Df(3R)Exel6198/shps*	78.3 ± 13.0 (14)	+++	95E5;95F8
*Df(3R)P3-4-7/shps*	16.8 ± 2.9 (10)	±	95F6-7;95F10
*jar/shps*	223.5 ± 9.6 (16)	+++	95F6-8
*crb/shps*	115.3 ± 12.8 (18)	+++	95F10-11
*jar/Df(3R)P3-4-7*	0 (3)	−	–
*crb/Df(3R)P3-4-7*	Lethal	N.A.	–
*GFP myosin VI/+*; *shps/TM3*	82.00 ± 26.01 (6)	N.D.	–
*GFP myosin VI/+*; *shps*	0.88 ± 0.64 (8)	N.D.	–
*shps/TM3* female × wild-type male	195.4 ± 3.6 (13)	+++	–
*shps* female × wild-type male	151.2 ± 5.4 (13)	+++	–

a Wild-type females were used for the crosses except for the crosses of *shps/TM3* female × wild-type male and *shps* female × wild-type male.

b Mean ± SE (*N*).

c +++, much sperm observed. ±, a few or no sperm observed. −, no sperm observed. N.A., not available. N.D., not determined.

d Breakpoints of each deficiency chromosome are shown. Gene spans, not breakpoints, are shown for *jar* and *crb*.

The hatchability of the eggs laid by females that copulated with wild-type or *shps/TM3* males was 92.9 and 92.0%, respectively. In contrast, the eggs laid by females mated with *shps* males hatched at a low rate (7.2%), and only eggs laid on d 1 and d 2 hatched. The hatch-to-adult rates were ∼95% for eggs from females mated to wild-type males (94.7%), and from those mated to *shps/TM3* males (95.7%). The hatch-to-adult rate from females mated to *shps* males were 75.0%. No adult flies were emerged from virgin females (0%). The mean numbers of progeny were 97.3 ± 13.2 (*N* = 28, wild type), 123.6 ± 13.1 (*N* = 28, *shps/TM3*), 1.3 ± 0.4 (*N* = 24, *shps*) and 0 (*N* = 25, virgin). Because heterozygous males were fertile, we concluded that the *shps* mutation is recessive for male sterility. Both the heterozygous and homozygous females were fertile (Table 1), indicating that *shps* is a strict male-sterile mutation. Male sterility of *shps* associated with low egg laying could be due to the inability of *shps* males to induce ovulation, and/or oviposition in the female. Another possibility is that low insemination rates and/or developmental failure following fertilization results in low hatchability. These possibilities are explored in the studies described below.

Other mutations that decrease the number of sperm in storage also result in decreased egg laying and hatchability (*e.g.*, *wst*, Ohsako and Yamamoto 2011). To further explore this possibility in *shps*, females mated to *shps/TM3*, and *shps* males were scored for sperm using lacto-acetic orcein to visualize sperm heads in the female seminal receptacle and spermathecae (Table 2). About 600 control sperm, compared to ∼40 from *shps* sperm, were found in female sperm storage organs 1 hr after copulation. After 24 hr, the number of sperm in both storage organs decreased. Since females begin to ovulate by 1.5 hr after copulation (Heifetz *et al.* 2000), the decreased sperm by 24 hr are thought to be used for fertilization in the 1st d after copulation. The decrease appeared to be greater in seminal receptacle (control: 275.1/437.1, 37.1% and *shps*: 3.7/31.1, 88% after 24 hr) than in spermathecae (control: 178.6/194.5, 8.2%; *shps*, 5.3/7.2, 26.4%). These results suggest that, in both control and *shps*, sperm stored in seminal receptacle were used at greater frequency than those in spermathecae. However, strikingly, the absolute number of *shps* sperm stored in the female sperm storage organs were far lower than the control. Therefore, all other considerations aside, the low numbers of offspring observed in the crosses with *shps* males (1.3 in *shps* *vs.* 123.6 in the control) can be linked back to the very low numbers of sperm stored in the female (38.3 in *shps* *vs.* 631.6 in the control, Table 2).

**Table 2:** Number of sperm stored in the female sperm storage organs

Male	Time After Copulation	Seminal Receptacle*^a^*	Spermatheca	(*N*)*^b^*
*shps/TM3*	1 hr	437.1 ± 20.6	194.5 ± 16.5	(9)
	24 hr	275.1 ± 14.3	178.6 ± 18.3	(12)
*shps*	1 hr	31.1 ± 16.0	7.2 ± 3.9	(12)
	24 hr	3.7 ± 1.4	5.3 ± 2.0	(12)

a Mean ± SE.

b Number of females dissected.

**Figure 4:**
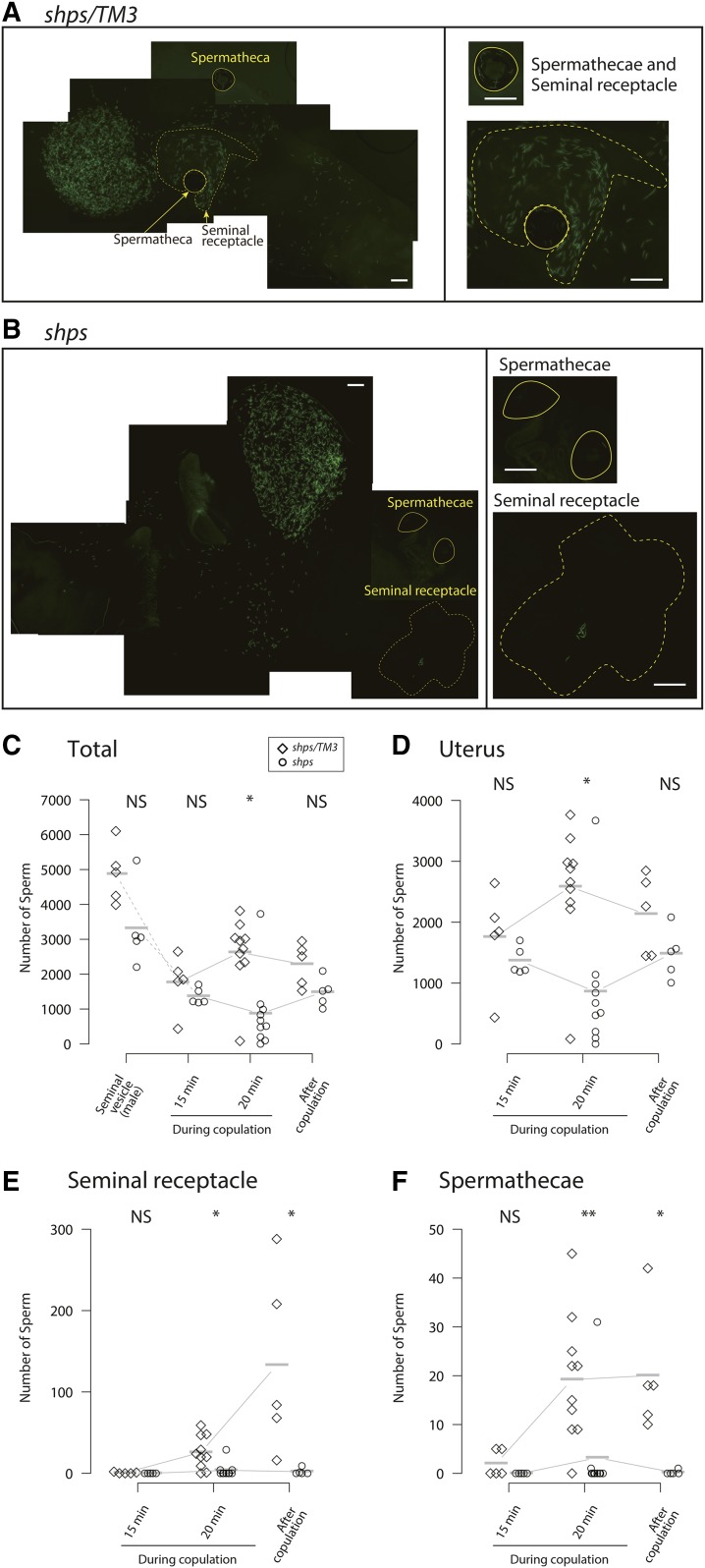
Sperm storage during and just after copulation finished. (A and B) Sperm head visualized by *protamineB-eGFP* (green) in uterus, seminal receptacle (dotted lines) and spermathecae (solid lines) 10 min after copulation finished. For the purpose of presentation, several photographs were merged by the method of maximum intensity Z-projection of ImageJ (Schneider *et al.* 2012). Bar, 50 μm. (C–F) Number of sperm visualized by *protamineB-eGFP* transgene in seminal vesicle of unmated males (*N* = 5), and those in females (uterus, seminal receptacle, and spermathecae) during copulation (ASM15 and ASM20; *N* = 5 and *N* = 10, respectively), and 10 min just after copulation finished (*N* = 5). Diamonds, *shps/TM3*; circles *shps*. NS, not significant; * significant at 5% level; ** significant at 1% level after Bonferroni correction. (C) Number of sperm in seminal vesicle of unmated males and those in females (uterus, seminal receptacle, and spermathecae). Wilcoxon-Mann-Whitney tests between *shps/TM3* and *shps* were as follows: *W* = 21, *P* = 0.09524 (seminal vesicle), *W* = 20, *P* = 0.1508 (15 min copulation), *W* = 83, *P* = 0.0115 (20 min copulation), *W* = 22, *P* = 0.05556 (just after copulation finished). (D) Number of sperm in uterus of females during copulation, and just after copulation finished. Wilcoxon-Mann-Whitney tests between *shps/TM3* and *shps* were as follows: *W* = 20, *P* = 0.1508 (15 min copulation), *W* = 83, *P* = 0.0115 (20 min copulation), *W* = 19, *P* = 0.222 (just after copulation finished). (E) Number of sperm in seminal receptacle during and just after copulation finished. There were no significant differences at 15 min during copulation and significant at 20 min during copulation and just after copulation finished (15 min copulation: *W* = 17.5, *P* = 0.4444, 20 min copulation: *W* = 86, *P* = 0.00408, just after copulation finished: *W* = 25, *P* = 0.00793). (F) Number of sperm in spermathecae during and just after copulation finished. Wilcoxon-Mann-Whitney tests between *shps/TM3* and *shps* were as follows: *W* = 17.5, *P* = 0.4444 (15 min copulation), *W* = 87, *P* = 0.00226 (20 min copulation), *W* = 25, *P* = 0.00793 (just after copulation finished).

Sperm begin to enter storage before the completion of copulation, suggesting the possibility that sperm, once stored, are discharged from the female sperm storage organs. There is precedence for sperm ejection from storage organs, as observed for *wst* sperm (Ohsako and Yamamoto 2011). Therefore, the low number of *shps* sperm in storage after 1 hr (Table 2) may either be due to (1) a high discharge rate from the sperm storage organs, or (2) *shps* sperm being impaired or prevented from entering the storage organs, or both. To distinguish between these two possibilities, sperm numbers during and following copulation were measured directly using a *protamineB-eGFP* transgene (Jayaramaiah Raja and Renkawitz-Pohl 2005) to visualize sperm heads in dissected female reproductive tracts (Figure 4). Similar numbers of sperm were observed in seminal vesicles of both *shps/TM3* and *shps* male before copulation (Figure 4C). Similarly, no significant differences were observed in the number of sperm transferred to the uterus by 15 min from the beginning of copulation (Figure 4D). At the time few, if any, sperm were observed in either the seminal receptacle (Figure 4E) or spermathecae (Figure 4F). However, dramatic differences in sperm storage patterns were observed beginning at 20 min from the beginning of copulation and a few min after copulation ends, where very few, if any, *shps* sperm were found in either the seminal receptacle or spermathecae (Figure 4, E and F). Comparison of the number of sperm in these early time points to those found 1 hr after copulation (Table 2) suggests that sperm begin to enter seminal receptacle and spermathecae as early as 15 min from the beginning of copulation and continue to enter storage for at least the next 24 hr. Therefore, we conclude that *shps* sperm are competent for transfer to females during copulation, but are severely impaired in the storage processes that follow.

### Sperm motion parameters

In mammals, hyperactivation is an essential prerequisite for sperm to acquire fertilization competency (Suarez 2008). Similar to mammalian sperm, *D. melanogaster* sperm also undergo hyperactivation with increased flagellar beat frequency after transfer to the uterus and before entrance into the sperm storage organs (Köttgen *et al.* 2011). To observe sperm motion, we used continuous photographs captured with a high-speed camera (300 frames/sec). When the control males (*shps/TM3*) were used, there were characteristic flagellum bend of sperm dissected from female uterus, whereas no characteristic flagellum bend was observed in sperm from male seminal vesicle (File S3). When *shps* males were used, beating sperm from the seminal vesicle and flagellum bend of sperm from uterus were also observed (File S3). The characteristic flagellum bend in female uterus was observed in sperm from males of both genotypes, suggesting that sperm of the control and *shps* males are hyperactivated, at least in some degree, after transfer to females from males.

We noticed that the motion of sperm from *shps* males seemed to be different from that of the control. To characterize sperm motion properties, three motion parameters were measured (Table 3); beat frequency and beat speed are sperm flagellum properties, and intensity index of sperm motion is a measurement of overall sperm motion, including sperm tail beat properties and other motion of whole sperm in the observation area *en masse*. Kruskal-Wallis test followed by multiple comparisons by Dunn’s methods (Zar 2010) showed that the three sperm motion parameters of the control after transfer to females were significantly higher than those of the control before transfer to females (Table 3). The beat frequency and the beat speed of sperm from *shps* males were intermediate. The intensity index of sperm from *shps* males before and after transfer to females did not differ from that of the control sperm after transfer to females (Table 3). These results are different from what we expected; the motion characteristic of sperm after transfer to females from *shps* males would be different from the control after transfer to females. However, taken together, sperm from *shps* males have characteristics of their motion that differ from those of the control. Although it seems that the parameters observed in our analysis did not provide a decisive proof to explain why sperm from *shps* males fail to be stored in the females, these results suggest that the sperm from *shps* males may have lost some type of function affecting their motion. It may be also explained that *shps* sperm are partly activated in the male before transfer to females, and are no longer activated in the uterus after transfer to females.

**Table 3:** Motion parameters of sperm before and after transfer to the females

Parameters and Genotype	Male Seminal Vesicles	Female Uterus
Beat frequency (Hz)		
* shps/TM3*	6.0 a ± 1.2 (10)	15.0 b ± 1.2 (9)
* shps*	9.3 a,b ± 1.3 (10)	11.8 a,b ± 1.9 (10)
	Kruskal-Wallis test *H* = 12.383, *df* = 3, *P* = 0.00618	
Beat speed (μm/s)		
* shps/TM3*	82.7 a ± 20.0 (10)	158.6 b ± 21.3 (9)
* shps*	106.8a,b ± 12.3 (10)	110.2 a,b ± 17.7 (10)
	Kruskal-Wallis test *H* = 9.9209, *df* = 3, *P* = 0.01925	
Intensity index		
* shps/TM3*	2.11 a ± 0.14 (30)	5.84 b ± 1.08 (6)
* shps*	3.36 b ± 0.28 (21)	6.31 b ± 0.88 (2)
	Kruskal-Wallis test *H* = 25.888, *df* = 3, *P* < 0.0001	

Mean ± SE (*N*). Means followed by the same lower case letters are not significantly different from each other by Dunn’s methods (*P* > 0.05) after Kruskal-Wallis test (see Table S1 in File S2).

### Sperm insemination ability and the female postmating responses are not affected by shps mutation

**Figure 5:**
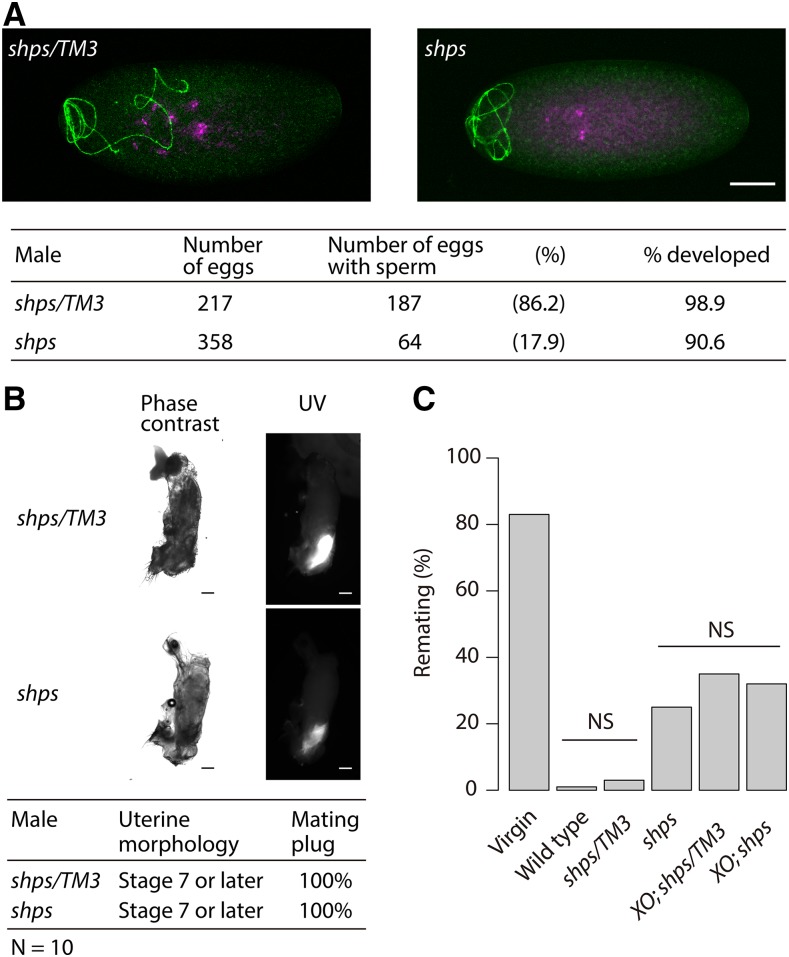
Insemination ability of *shps* sperm and female postmating responses. (A) The eggs inseminated with *shps* sperm initiated normal development. Sperm detected by a DROP1.1 antibody and visualized with Alexa Fluor 488 (green) and nuclei by DAPI (magenta) are merged. Eggs laid by the female that copulated with a *shps/TM3* male or a *shps* male were collected within 5 hr of oviposition. Bar, 50 μm. (B) Uterine morphological conformation and mating plug formation of the females that copulated with *protamineB-eGFP/+*; *shps/TM3* (*shps/TM3*) or *protamineB-eGFP/+*; *shps* (*shps*) male (ASM 20). The conformational stages of uteri (Adams and Wolfner 2007; Avila and Wolfner 2009) were stage 7 or later. All females showed a signal of autofluorescence of PEB-me—a major component of the mating plug. *N* = 10. Bar, 100 μm. (C) Remating frequency. Number of females mated with wild-type males were counted a day after first mating. Wild-type, *shps/TM3*, *shps*, *XO*; *shps/TM3*, *XO*; *shps* females that were mated a day before the remating test were used. Unmated females were used as controls (Virgin); these females mated first time in this test, that is, it is not remating. Efficiency of suppressing remating by seminal fluid from *shps* males were comparable with that from spermless (XO) males. $\chi_{5}^{2}$ = 211.7, *P* < 0.001, chi-square test for contingency table. NS, not significant by pairwise comparisons with sequential Bonferroni correction. *N* = 100.

In addition to the very low numbers of sperm stored in females, the low hatchability seen in crosses with *shps* males could be due to a failure in embryonic development postfertilization. To examine this possibility, eggs fertilized by *shps* sperm were observed using a sperm-specific antibody (Graner *et al.* 1994) and a DNA-specific dye to monitor fertilization and early development. Although females mated to *shps* males oviposited normal levels of eggs compared to controls in d 1 (Figure 3A), only 17.9% (64/358) eggs were fertilized and initiated mitotic divisions as compared to over 85% (187/217) in the control crosses (Figure 5A). These results are consistent with the low numbers of *shps* sperm in storage (Table 2), and demonstrate that the *shps* are fertilization-competent. These results are also consistent with the low viability of *shps* crosses where viable adults did not emerge in the first few days after egg laying (Figure 3), presumably due to depletion of sperm in storage. The large number of unfertilized eggs is most likely due to both the decrease in availability of sperm and the induced oviposition following copulation and the introduction of seminal fluids.

There were no differences in the conformational changes of the uteri between the females copulated with *shps/TM3* males and those with *shps* males (Figure 5B). All females showed the stage 7 or later uteri at ASM 20, whereas the uteri of the females copulated with males lacking Acp36DE reach stage 7 (Adams and Wolfner 2007; Avila and Wolfner 2009). Therefore, the uterus conformational modification function of Acp36DE is not affected by the *shps* mutation.

Mating plug formation, as measured by autofluorescence (Lung and Wolfner 2001), was also not affected by crosses with *shps* males (Figure 5B). Therefore, *shps* does not appear to affect the transfer of ejaculatory bulb proteins from males to females and mating plug formation in the uterus. Reduced receptivity, one of the female postmating responses, was also monitored following matings of wild-type females to *XO*; *shps* males that lack sperm but produce seminal fluids (Figure 5C). As expected, both wild-type and *shps/TM3* males repress remating at similar rates, while no differences were observed in crosses with *shps*, *X/O*; *shps/TM3* or *X/O*; *shps* males. Thus, female remating is suppressed both in the presence or absence of *shps* sperm, consistent with the notion that *shps* has no effect on female postmating responses. Finally, we note that *shps* males are not as effective in suppressing female remating (∼30–35%) as the wild-type (∼3–5%). Sex-peptide, one of seminal fluid proteins, binds to sperm (Peng *et al.* 2005), and induces several female postmating responses, such as increased oviposition and reduced receptivity (Aigaki *et al.* 1991). Although sperm from *shps* males were transferred to females, only a few were stored. Females lost most sperm, and also sex-peptide associated with them, within a few hours of copulation. This might be a reason why suppression of remating by the sperm effect was not observed in females that copulated with *shps* males. Loss of sperm with functional sex-peptide might also cause reduced egg laying in the females copulated with *shps* males (Figure 3A).

### The shps gene is CG13611, which encodes a flavin-containing amine oxidase with a C-terminal transmembrane region

**Figure 6:**
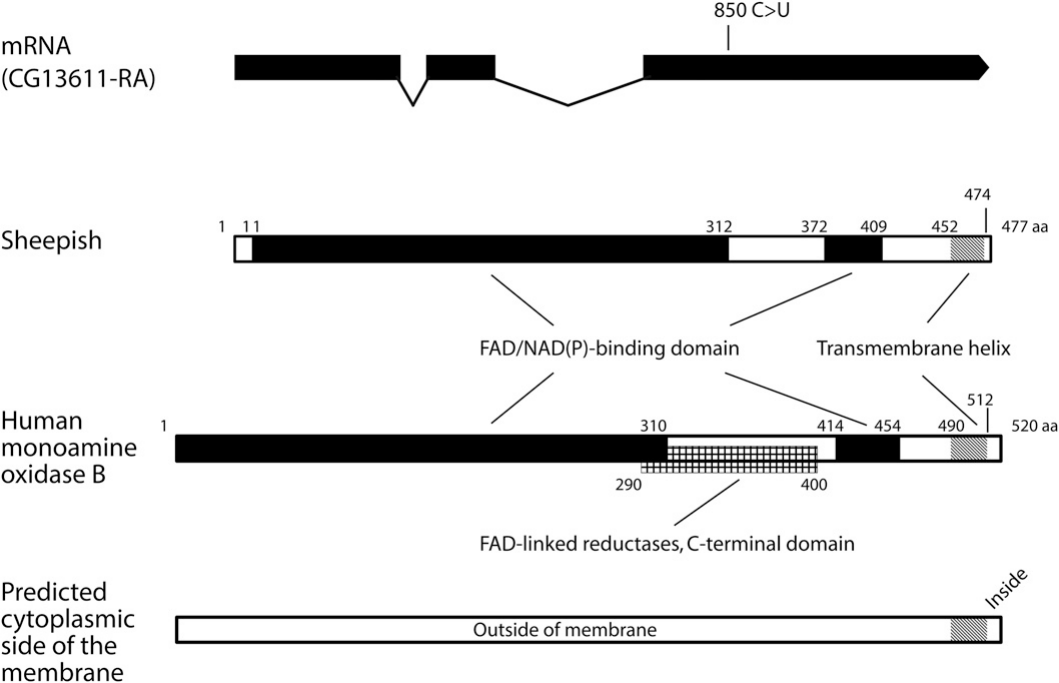
A nucleic substitution of *shps* mRNA and the estimated structure of Shps protein. CG13611-RA is an mRNA registered in FlyBase (Gramates *et al.* 2017). In *shps* mutants, cytosine at position 850 is substituted with uracil, resulting in glutamine being replaced by a termination codon. A FAD/NAD(P)-binding domain and its boundaries were predicted using Superfamily 1.75 (Gough *et al.* 2001), and a transmembrane helix region and its boundaries were predicted using TMHMM 2.0 (Sonnhammer *et al.* 1998; Krogh *et al.* 2001). Human MAOB (predicted amino acid sequence was obtained from Ensembl, Yates *et al.* 2016) is presented as a reference. Superfamily 1.75 predicted a FAD/NAD(P)-binding domain and also FAD-linked reductases, C-terminal domain, the latter of which was not predicted in Shps (this domain was predicted in the homologous protein of *D. willistoni*; see Figure S2 in File S2). Cytoplasmic side of the membrane predicted by TMHMM 2.0 is shown schematically at the bottom.

Recombination mapping using a multiple marker strain revealed that *shps* is located at 3–84, which corresponds to ∼95F of the right arm of the third chromosome (Map Conversion Table obtained from FlyBase; Gramates *et al.* 2017). The deficiency mapping showed that the *shps* locus was mapped in the interval between *jar* [the right end of *Df(3R)Exel6198* and the left end of *Df(3R)Exel6199* and *Df(3R)Exel8178*) and *crb* (the right end of *Df(3R)P3-4-7*] (Figure S1A in File S1 and Table 1). Within the interval, there are seven protein coding genes (FlyBase, Gramates *et al.* 2017): *Orct2*, *Orct*, *CG13611*, *CG6356*, *CG34290*, *CG6364*, and *CG5715* (Figure S1A in File S1). Since, among these, *CG13611* is recorded as testis-specific (Parisi *et al.* 2004; Chintapalli *et al.* 2007; Graveley *et al.* 2011), we thought it the most probable candidate to be responsible for the male sterile mutation, *shps*. The genome region of *CG13611* was sequenced (DDBJ/GenBank/EMBL accession No. AB932860) and it was found that a nucleotide of the third exon of *CG13611* of *shps* mutant strain was substituted (Figure 6) and estimated to result in a nonsense mutation.

The wild-type allele of *CG13611* was cloned into the rescue construct vector *pUAST-attB-loxP*, and two transformant lines were independently established [*PBac{CG13611${}^{+}$}VK00037 (S2)* and *(S5)*]. Because the insertion locations of the transgenes were identical, no position effects were expected between the lines. Table 4 shows that both lines rescued the sterility of the *shps* mutation in the two different mutant backgrounds [homozygous for *shps* and hemizygous *shps/Df(3R)P3-4-7*]. Since there were no differences between the numbers of progeny of the lines with a transgene in the mutant background, and those in the control background (heterozygous for *shps*), the transgene of the wild-type *CG13611* allele completely rescued the sterility of *shps*. We concluded that *shps* is encoded by *CG13611*.

**Table 4:** Number of progeny from rescue experiment

Male*^a^*	Number of Progeny*^b^*
*PBac{CG13611${}^{+}$}VK00037 (S2)/+; shps/TM3*	180.6 ± 9.6 (25)
*+*; *shps*	0.8 ± 0.5 (26)
*PBac{CG13611${}^{+}$}VK00037 (S2)/+; shps*	183.9 ± 9.4 (25)
*PBac{CG13611${}^{+}$}VK00037 (S5)/+; shps/TM3*	168.1 ± 8.1 (23)
*+*; *shps*	0.6 ± 0.4 (23)
*PBac{CG13611${}^{+}$}VK00037 (S5)/+; shps*	168.7 ± 9.3 (26)
*PBac{CG13611${}^{+}$}VK00037 (S2)/+; shps/TM3*	152.1 ± 12.3 (17)
*+*; *shps*	5.2 ± 1.9 (26)
*PBac{CG13611${}^{+}$}VK00037 (S2)/+; shps/Df(3R)P3-4-7*	166.1 ± 10.2 (20)
*PBac{CG13611${}^{+}$}VK00037 (S5)/+; shps/TM3*	163.1 ± 9.8 (22)
*+*; *shps*	0.2 ± 0.1 (26)
*PBac{CG13611${}^{+}$}VK00037 (S5)/+; shps/Df(3R)P3-4-7*	157.9 ± 10.6 (27)

a Three lines of males grouped together are sibs that emerged from the same parents. Two independently established lines, S2 and S5, were tested.

b Mean ± SE (*N*).

Orthologous proteins of *shps* are found widely in animals, such as, insects, polychaeta, cephalochordates, and agnatha (OrthoDB; Waterhouse *et al.* 2011). The structure of the predicted protein, Shps, is shown in Figure 6. A FAD/NAD(P)-binding domain and a transmembrane helix region in the C-terminal were predicted using Superfamily 1.75 (Gough *et al.* 2001) and transmembrane hidden Markov model (TMHMM) 2.0 (Sonnhammer *et al.* 1998; Krogh *et al.* 2001), respectively. This structure, a FAD/NAD(P)-binding domain with a C-terminal transmembrane region, is found in other 11 species of *Drosophila* (Figure S2 in File S2), and is also found in mammalian monoamine oxidase B (Mitoma and Ito 1992; Binda *et al.* 2002). Using HMMER protein homology search (Finn *et al.* 2015), we also found that, although it is slightly too big, the *E*-value (0.0077) of monoamine oxidase B is the smallest within human proteins in response to a query of the predicted Shps protein sequence. Therefore, we concluded that Shps is a monoamine oxidase-like protein.

## Discussion

The *shps* gene was first isolated in screens for male sterility as previously reported (Ohsako *et al.* 2003; Hirai *et al.* 2004; Ohsako and Yamamoto 2011). Genome-wide surveys recorded *shps* as one of the testis-specific expression genes (Parisi *et al.* 2004; Chintapalli *et al.* 2007; Graveley *et al.* 2011), with little or no expression detected in the male accessory gland and the female reproductive tract (Lawniczak and Begun 2004; McGraw *et al.* 2004; Prokupek *et al.* 2009). Although its expression is testis-specific (Parisi *et al.* 2004; Chintapalli *et al.* 2007; Graveley *et al.* 2011), the function of *shps* does not appear to affect spermatogenesis *per se*, as sperm production appears normal in any way that our analyses could detect. Indeed, proteome analyses of sperm and testes did not detect *shps* products (Dorus *et al.* 2006; Takemori and Yamamoto 2009; Wasbrough *et al.* 2010; Yamamoto and Takemori 2010), probably due to the smaller amounts of the protein compared with other testis-expressed proteins. Therefore, there is, at present, no evidence for Shps protein in the mature sperm. Previous studies have clearly shown a major role for seminal fluid proteins in male fertility, including sperm storage and utilization. Seminal fluid proteins secreted by the accessory glands mix with sperm during copulation and transfer to females (for review, see Avila *et al.* 2011). Although over a dozen seminal fluid proteins have been identified to date, only two have been shown to affect sperm storage and release (Avila *et al.* 2010; Bloch Qazi and Wolfner 2003). Here, we examined the functional implications of *shps* for male reproduction. Our results show that *shps* sperm are effectively transferred to the female during copulation but do not enter sperm storage efficiently. Therefore, fewer sperm are available for fertilization, thus significantly reducing overall male fertility. Our results further show that *shps* encodes a putative flavin-containing amine oxidase with a C-terminal transmembrane region. Taken together, the testis-specific expression and functional category of *shps* suggest a new class of male sterile mutants affecting sperm storage in females.

Although present in large numbers in the uterus following copulation, very few *shps* sperm were observed in the seminal receptacle and spermathecae, suggesting an impaired ability of sperm to enter the storage organs. The pattern of sperm utilization of *shps* phenotype differs significantly from that observed in sperm carrying mutations in the *wst* gene (Ohsako and Yamamoto 2011). *wst* sperm are rapidly discharged from the uterus following copulation and ovulation, resulting in a severe reduction in sperm numbers in sperm storage organs. In contrast, *shps* sperm were as motile as the control before transfer to a female, but were not efficiently stored in the sperm storage organs. In wild-type sperm, an increase in sperm motion parameters following transfer to females was observed, whereas characteristics of sperm motion parameters of sperm from *shps* males were different from the control, suggesting that *shps* mutation has some effect on sperm motion. The relationship between female sperm storage and sperm motion properties in *shps* sperm remains to be clarified by further studies. A few progeny of *shps* males emerged from the eggs laid a few days after copulation, and no hatched eggs were laid on the 4th d after copulation or later. Most eggs inseminated with sperm from *shps* males initiated development, suggesting that unhatched eggs were not inseminated, similar to those laid by unmated females. These observations indicate that the *shps* sperm that occasionally enter the sperm storage organs in females have the ability to fertilize eggs, and are used for fertilization by the female in the early term of her egg laying period. We conclude that the cause of the sterility of *shps* males is the loss of the ability of sperm to enter the sperm storage organs of females in the uterus.

The *shps* gene encodes a putative flavin-containing amine oxidase or monoamine oxidase. The predicted structure of the Shps protein consists of a catalytic domain with a transmembrane region at the C-terminal end. This structure is also found in orthologs of other 11 species of *Drosophila* (Figure S2 in File S2), suggesting that the structure of Shps is conserved in the genus *Drosophila*. Shps is different from previous reported mutation concerning sperm storage, *kl-1* (WD40-rich proteins, Kiefer 1969; Vibranovski *et al.* 2008), *Pkd2* (a cation channel protein, TRPP2, Gao *et al.* 2003; Watnick *et al.* 2003; Köttgen *et al.* 2011; Yang and Lu 2011), and *lobo* (a protein associated with outer doublet microtubules of flagellum, Yang *et al.* 2011). Shps has a general resemblance to human monoamine oxidase B (MAOB), which has a catalytic domain with a C-terminal transmembrane region. Monoamine oxidases produce reactive oxygen species (ROS) through the reduction of molecular oxygen to hydrogen peroxide (Di Lisa *et al.* 2009). Although ROS have a negative effect, such as oxidative stress damaging sperm, they contribute positively to sperm regulation (Aitken 2000; Aitken *et al.* 2012). MAOB, an activity of which in mitochondrial function is thought to be related to Parkinson’s disease (Cohen *et al.* 1997; Cohen and Kesler 1999; Di Lisa *et al.* 2009; Jenner 2012), binds to the outer membranes of mitochondria (Mitoma and Ito 1992) and is widely distributed among tissue types (Schnaitman *et al.* 1967). Kiefer (1969) discussed that a defect in mitochondrial function might be the reason structurally normal and motile *kl-1* sperm failed to be stored in females. It is possible that mitochondria play a crucial role in sperm storage in *Drosophila*. It will be of interest to determine the subcellular location of Shps, especially at the sperm plasma membrane or mitochondria, during spermatogenesis and sperm maturation.

The number of sperm stored in the seminal receptacle was reduced more rapidly than that in spermathecae (Table 2), which is consistent with the notion that the seminal receptacle is the sperm storage organ primarily used in *D. melanogaster* (Miller 1950; Pitnick *et al.* 1999). Declines in hatchability on d 9 and 10 after copulation were observed in the controls (Figure 3). It is likely that the number of sperm possessed by the female is then insufficient for fertilization. Sperm effects on female postmating responses relating to sex-peptide (Aigaki *et al.* 1991) were not detected in the females copulated with *shps* males. This can be explained by the fact that females store only a few sperm, with which sex-peptide associated. The females that copulated with *shps* males stored a few sperm and laid uninseminated eggs. Ovulation and oviposition are induced by seminal fluid proteins even if the female does not have sperm (Neubaum and Wolfner 1999; Bloch Qazi and Wolfner 2003; Wolfner 2009). The many uninseminated eggs laid by the females that copulated with *shps* males on d 1–3 are likely due to this seminal fluid effect. Observation of uterine morphological conformation change, mating plug formation, and a remating test suggest that female postmating responses is functional for phenotypes not related to sperm storage.

## Supplementary Material

Supplemental material is available online at www.g3journal.org/lookup/suppl/doi:10.1534/g3.117.300171/-/DC1.
