# Supplementary material for: Severe Fertility Effects of sheepish Sperm Caused by Failure To Enter Female Sperm Storage Organs in Drosophila melanogaster
Source: G3 (Bethesda). 2017 Nov 20;8(1):149–60. doi: 10.1534/g3.117.300171 (PMC5765343; doi:10.1534/g3.117.300171)
Supplement: Supplementary file 1 [file 149FileS1.pdf]

Supplemental File S1

Severe fertility effects of *sheepish* sperm caused by failure to enter female sperm storage organs in *Drosophila melanogaster*

Masatoshi Tomaru, Takashi Ohsako, Masahide Watanabe,
Naoto Juni, Hiroshi Matsubayashi, Hiromi Sato,
Ayako Takahashi and Masa-Toshi Yamamoto

Department of Drosophila Genomics and Genetic Resources,
Center for Advanced Insect Research Promotion,
Kyoto Institute of Technology,
Kyoto 616-8354,
Japan

Determination of break point ends of deficiency, *Df(3R)P3-4-7, P{lacW}degenerated*

Materials and Methods

A new strain, designated as P3-4-7, was established from the progeny of the cross of *y* w 1118; P{lacW}crb j1B5/TM3, Sb* and *w*; Dr/TMS, P{Δ2-3}99B*. Deep-red-eyed progeny were selected to obtain flies that were expected to have two insertions, an original and a new one, since *P* elements frequently transpose to sites nearby (Tower *et al.* 1993).

The genomic DNA surrounding the insertion of P3-4-7 was cloned by inverse PCR (http://www.fruitfly.org/about/methods/inverse.pcr.html). DNA isolated from P3-4-7 males was digested using *Sau*3A I restriction enzyme. After inactivation of the enzyme, restriction fragments were ligated for 18 h in the presence of T4 DNA ligase and precipitated. DNA was PCR amplified using 5' end primer sets (Plac1: 5'-CACCCAAGGCTCTGCTCCCACAAT-3' and Plac4: 5'-ACTGTGCGTTAGGTCCTGTTCATTGT-3') or 3' end primer sets (Pry1: 5'-CCTTAGCATGTCCGTGGGGTTTGAAT-3' and Pry4: 5'-CAATCATATCGCTGTCTCACTCA-3') with Ex Taq DNA polymerase (TAKARA). The amplified fragments were sequenced and the obtained sequences were compared to the Drosophila Genome Database using FlyBase BLAST (http://flybase.org/blast/).

Since P3-4-7 has deep red eyes, it was expected that P3-4-7 has two *P{lacW}* insertions. To detect each end of the two insertions, eight PCR primer sets consisting of 10 primers were designed. The primers are as follows:
3P (5'-CCACGGACATGCTAAGGGTTAATC-3'),
5P (5'-CGCACACAACCTTTCCTCTCAACA-3'),
a (5'-TATAAGGGTGCTGGGTGAGT-3'),
b (5'-GCTTGCGATGATGCTGAACT-3'),
c (5'-TGGACTGAAACATAGCCACGAG-3'),
d (5'-GTTTTCCTACTACCCTCACCCT-3'),
e (5'-CTTTGATCTCGTAGGGCACAAC-3'),
f (5'-GTGCTGCCTGTTTATGCTGAGTTC-3'),
g (5'-ATGCGACGTAATTTGAGCTGCG-3') and
h (5'-CATCTTCGCTCTCTCCTCCTGAATG-3'). DNA extracted from P3-4-7 or *P{lacW}crb j1B5* strain was PCR-amplified using the above eight primer pairs. The amplified fragments were detected by agarose gel electrophoresis.

Results

Since the male heterozygotes of *jar/shps* and *crb*^{j1B5}*/shps* were fertile (Table 1), *shps* is not an allele of *jar* or *crb*. To obtain a new insertion allele of *shps*, a *P{lacW}* element of *P{lacW}crb*^{j1B5} strain was remobilized using a *Δ2-3* transposase source. *P{lacW}* has high probability of transposing to a site near its original insertion site (Tower *et al.* 1993). The deep-red-eyed strain, P3-4-7, obtained in this study, was expected to have two *P{lacW}* insertions, the original *P{lacW}crb*^{j1B5} and a new one. Since P3-4-7 did not complement the sterility of *shps* and the lethality of *crb* (Table 1), we thought that the new *shps* mutant allele is due to the second insertion of *P{lacW}* and the original insertion remained in the chromosome. Inverse PCR revealed that the 5' end of the P3-4-7 insertion was the same as the original one (Figure S1A, its sequence is DDBJ/GenBank/EMBL accession No. AB932858) and the 3' end of the P3-4-7 insertion is located in the intron of *jar* (Figure S1A, its sequence is DDBJ/GenBank/EMBL AB932859). P3-4-7 did not complement the sterility of *jar* (Table 1). It has, thus, also a mutant allele of *jar*. *GFP-myosin VI* expresses full-length Jar tagged with GFP in the testis and rescues the sterility of *jar* (Noguchi *et al.* 2006). It would also rescue the sterility of *shps*, if *shps* is an allele of *jar*. However, *GFP-myosin VI/+; shps* males were sterile (Table 1), indicating that *GFP-myosin VI* does not rescue the sterility of *shps*.

Inverse PCR analysis on P3-4-7 detected no 3' end sequence of the original insertion. It is possible that P3-4-7 is not a line with two *P{lacW}* insertions, but that a chromosomal segment between the two insertions in *jar* and *crb* is deleted. To examine this possibility, eight PCR primer sets were designed to detect each end of two insertions (Figure S1A) and PCR amplification was performed on P3-4-7 and *P{lacW}crb*^{j1B5}. From *P{lacW}crb*^{j1B5}, only four fragments in *crb* would be amplified. If P3-4-7 is a double-insertion strain, it is expected that eight different fragments would be amplified by all eight primer sets in *jar* or *crb*. In contrast, if P3-4-7 is a deficiency, four outer fragments would be amplified but internal fragments would not. As expected, from the original *P{lacW}crb*^{j1B5} line, no PCR fragments were detected using primer sets to detect the *jar* region (Figure S1B), whereas the four fragments in *crb* with expected lengths were amplified (Figure S1C). From P3-4-7, only four outer fragments were amplified, but no internal ones were (Figures S1B and S1C), suggesting a deficiency. Therefore, P3-4-7 was revealed to be not a simple double-insertion strain but a deficiency lacking a chromosomal region between *jar* and *crb*. We thus designated this deficiency as *Df(3R)P3-4-7, P{lacW}degenerated*, of which the deleted chromosomal segment

is about 31.6 kb, spanning from *jar* and *crb* (95F6-7;95F10). This is a plausible result explaining the lack of complementation by *Df(3R)P3-4-7* of the sterility of *shps* and *jar* and the lethality of *crb*, and the unrescued sterility of *shps* with *GFP-myosin VI* (Table 1).

Literature Cited

Noguchi, T., M. Lenartowska, and K. G. Miller, 2006 Myosin VI stabilizes an actin network during *Drosophila* spermatid individualization. Mol. Biol. Cell **17**: 2559–2571.

Tower, J., G. H. Karpen, N. Craig, and A. C. Spradling, 1993 Preferential transposition of Drosophila *P* elements to nearby chromosomal sites. Genetics **133**: 347–359.

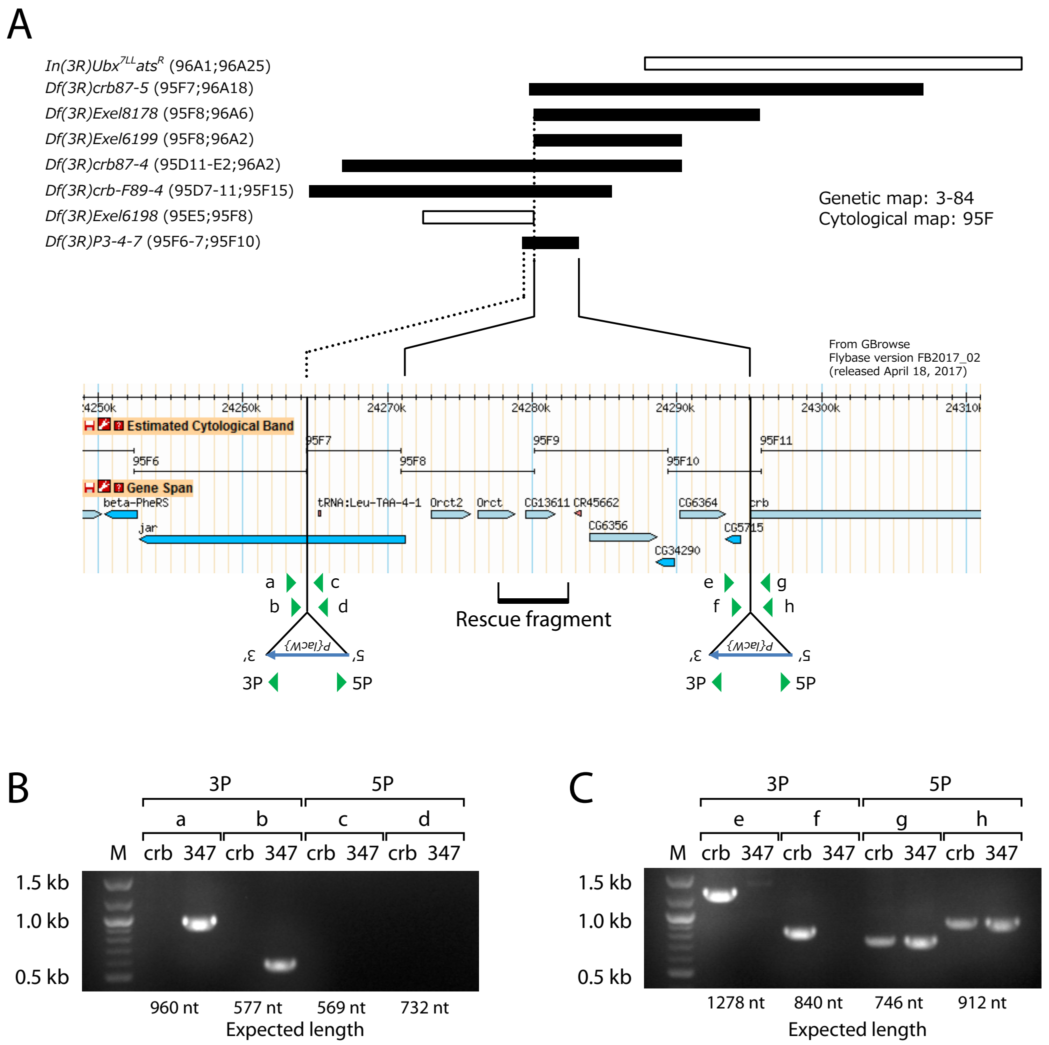
A
In(3R)Ubx7LLatsR (96A1;96A25)
Df(3R)crb87-5 (95F7;96A18)
Df(3R)Exel8178 (95F8;96A6)
Df(3R)Exel6199 (95F8;96A2)
Df(3R)crb87-4 (95D11-E2;96A2)
Df(3R)crb-F89-4 (95D7-11;95F15)
Df(3R)Exel6198 (95E5;95F8)
Df(3R)P3-4-7 (95F6-7;95F10)
Genetic map: 3-84
Cytological map: 95F
From GBrowse
Flybase version FB2017_02
(released April 18, 2017)
Estimated Cytological Band
Gene Span
Rescue fragment
3P
5P
B
3P
5P
M crb 347 crb 347 crb 347 crb 347
1.5 kb
1.0 kb
0.5 kb
960 nt
577 nt
569 nt
732 nt
Expected length
C
3P
5P
M crb 347 crb 347 crb 347 crb 347
1.5 kb
1.0 kb
0.5 kb
1278 nt
840 nt
746 nt
912 nt
Expected length

Figure S1.

(A). Schematic map of deficiencies used for mapping and a genome map. Filled boxes are deficiencies that did not complement the sterility of *shps* and open boxes are deficiencies that did complement it (Table 1). Although P3-4-7 was a line expected to have two *P{lacW}* insertions, it was a deficiency. It is shown as *Df(3R)P3-4-7*. Below is a genome map obtained from GBrowse at FlyBase (version FB2017_02, released April 18, 2017). A *P{lacW}* insertion of original crb^{j1B5} and the expected second insertion of P3-4-7 are superimposed on the map. Triangles, right-pointing (a, b, e, f and 5P) and left-pointing (c, d, g, h and 3P), are schematic representations of PCR primers. A genomic fragment used for the rescue experiment is shown below the genome map. (B and C). Detection of PCR amplification from P3-4-7 (347) and *P{lacW}*crb^{j1B5} (crb) flies. The primer pairs 'a and 3P' and 'b and 3P' are for the 3' end of the new insertion and the expected amplified fragment lengths are 960 nt and 577 nt, respectively. The primer pairs 'c and 5P' (569 nt) and 'd and 5P' (732 nt) are for the 5' end of the new insertion, the primer pairs 'e and 3P' (1278 nt) and 'f and 3P' (840 nt) are for the 3' end of the original insertion *P{lacW}*crb^{j1B5} and the primer pairs 'g and 5P' (746 nt) and 'h and 5P' (912 nt) are for the 5' end of the original insertion. Lanes corresponding to each primer set are shown above the electrophoresis and the expected fragment lengths are shown below. Lane M is a size marker ladder.
