# Supplementary material for: Severe Fertility Effects of sheepish Sperm Caused by Failure To Enter Female Sperm Storage Organs in Drosophila melanogaster
Source: G3 (Bethesda). 2017 Nov 20;8(1):149–60. doi: 10.1534/g3.117.300171 (PMC5765343; doi:10.1534/g3.117.300171)
Supplement: Supplementary file 2 [file 149FileS2.pdf]

Supplemental File S2

Severe fertility effects of *sheepish* sperm caused by failure to enter female sperm storage organs in *Drosophila melanogaster*

Masatoshi Tomaru, Takashi Ohsako, Masahide Watanabe,
Naoto Juni, Hiroshi Matsubayashi, Hiromi Sato,
Ayako Takahashi and Masa-Toshi Yamamoto

Department of Drosophila Genomics and Genetic Resources,
Center for Advanced Insect Research Promotion,
Kyoto Institute of Technology,
Kyoto 616-8354,
Japan

Supplemental File S2 contains as follows:

- Figure S2
- Figure S3
- Table S1
- Table S2
- Supplemental File Legend (Supplemental File S3)

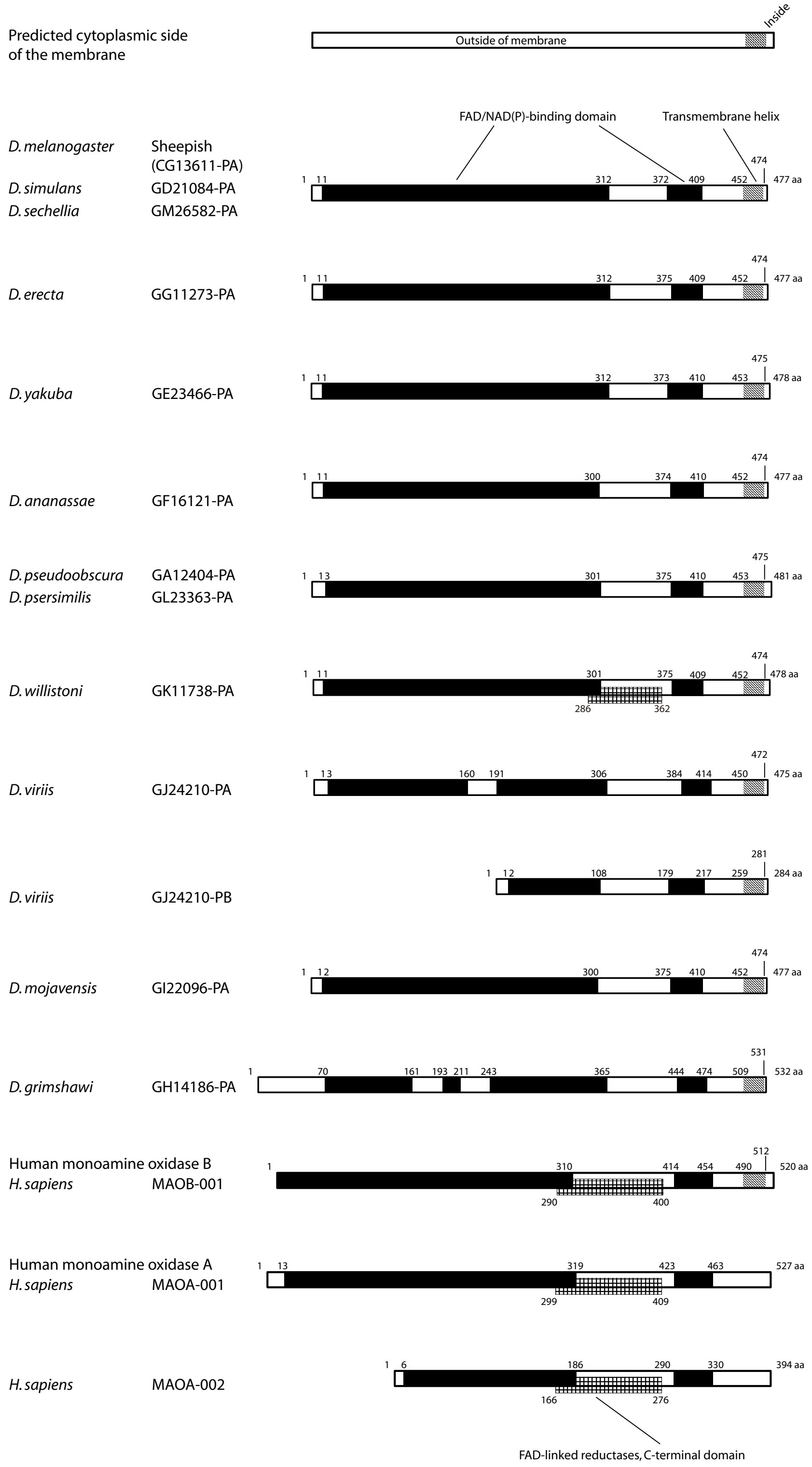

Predicted cytoplasmic side of the membrane
Outside of membrane
Inside
FAD/NAD(P)-binding domain
Transmembrane helix
D. melanogaster Sheepish (CG13611-PA)
D. simulans GD21084-PA
D. sechellia GM26582-PA
1 11 312 372 409 452 474 477 aa
D. erecta GG11273-PA
1 11 312 375 409 452 474 477 aa
D. yakuba GE23466-PA
1 11 312 373 410 453 475 478 aa
D. ananassae GF16121-PA
1 11 300 374 410 452 474 477 aa
D. pseudoobscura GA12404-PA
D. psersimilis GL23363-PA
1 13 301 375 410 453 475 481 aa
D. willistoni GK11738-PA
1 11 301 375 409 452 474 478 aa
286 362
D. viriis GJ24210-PA
1 13 160 191 306 384 414 450 472 475 aa
D. viriis GJ24210-PB
1 12 108 179 217 259 281 284 aa
D. mojavensis GI22096-PA
1 12 300 375 410 452 474 477 aa
D. grimshawi GH14186-PA
1 70 161 193 211 243 365 444 474 509 531 532 aa
Human monoamine oxidase B
H. sapiens MAOB-001
1 310 414 454 490 512 520 aa
290 400
Human monoamine oxidase A
H. sapiens MAOA-001
1 13 319 423 463 527 aa
299 409
H. sapiens MAOA-002
1 6 186 290 330 394 aa
166 276
FAD-linked reductases, C-terminal domain

Figure S2. Protein structures of Shps and its homologous proteins of 12 species of *Drosophila* (predicted amino acid sequences were obtained from FlyBase, Gramates *et al.* 2017 FlyBase at 25: Looking to the future. Nucleic Acids Res. **45:** D663–D671) and human monoamine oxidases (predicted amino acid sequences were obtained from Ensembl, Yates *et al.* 2016 Nucleic acids research **44:** D710–D716), with domains predicted by Superfamily 1.75 (Gough *et al.* 2001 Assignment of homology to genome sequences using a library of hidden Markov models that represent all proteins of known structure. J. Mol. Biol. **313:** 903–919) and a transmembrane helix region predicted by TMHMM 2.0 (Sonnhammer *et al.* 1998 A hidden Markov model for predicting transmembrane helices in protein sequences. Proceedings of the Sixth International Conference on Intelligent Systems for Molecular Biology **6:** 175–182; Krogh *et al.* 2001 Predicting transmembrane protein topology with a hidden Markov model: Application to complete genomes. J. Mol. Biol. **305:** 567–580). Cytoplasmic side of the membrane predicted by TMHMM 2.0 is schematically shown on the top. All 12 species of *Drosophila* have a FAD/NAD(P)-binding domain that is outside of the membrane and a transmembrane helix at the C-terminal end. Its structure is similar to human monoamine oxidase B (MAOB). Human MAOs have FAD-linked reductases, C-terminal domain, whereas only *D. willistoni* has this domain with a low E-value (P = 0.0000338) in *Drosophila*. Species with FAD-linked reductases, C-terminal domain of which an E-value greater than 0.0001 (weak HMM library classifications) are as follows: *D. ananassae* (P = 0.00275), *D. pseudoobscura* (P = 0.0745), *D. persimilis* (P = 0.0745), *D. virilis* GJ24210-PA (P = 0.0667), *D. virilis* GJ24210-PB (P = 0.00275), *D. mojavensis* (P = 0.00051), *D. grimishawi* (P = 0.00706). Protein sequence alignment is in Figure S3.

CLUSTAL O(1.2.4) multiple sequence alignment

```
FBpp0083984      -----------------------------------------------------------M   1
FBpp0219486      -----------------------------------------------------------M   1
FBpp0208059      -----------------------------------------------------------M   1
FBpp0129819      -----------------------------------------------------------M   1
FBpp0268476      -----------------------------------------------------------M   1
FBpp0119313      -----------------------------------------------------------M   1
FBpp0283967      ---------------------------------------------------------MDQ   3
FBpp0187470      ---------------------------------------------------------MDQ   3
FBpp0240881      -----------------------------------------------------------M   1
FBpp0238627      -----------------------------------------------------------M   1
FBpp0392421      ------------------------------------------------------------   0
FBpp0171313      -----------------------------------------------------------M   1
FBpp0148092      MEKAKKMEVEMMEVRRRRRRGRGLKGEADEEGDRDGEGNQEGAGERERNTKRDGDGQDYM   60
MAOB-001         ------------------------------------------------------------   0
MAOA-001         ---------------------------------------------------------MEN   3
MAOA-201         ------------------------------------------------------------   0

FBpp0083984      DLPFGTQTPELDVLIVGGGLSGLASALKILAMESTLKVRMIEASDALGGLMGQN------   55
FBpp0219486      DLPFGTQTPELDVLIVGGGLSGLSSALKILALESTLKVRMIEASDGLGGLMGQN------   55
FBpp0208059      DLPFGTQTPELDVLIVGGGLSGLSSALKILALESTLKVRMIEASDGLGGLMGQN------   55
FBpp0129819      DLPFGTQTPELDVLIVGGGLSGLASALKILSLESTLKVRIIEASDGLGGLMGQN------   55
FBpp0268476      DLPFGTQTPELDVLIVGGGLSGLTSALKILAMESTLKVRLIEASDGLGGQMGQN------   55
FBpp0119313      DLPFGNQTPELDVLIVGAGLSGLSSAVKILAKESTLRLRVIESNETLGGQLGEN------   55
FBpp0283967      HLPFGTEQPELDVVIVGSGLSALTSAVKLLAKETTLHIRILDENSRPGGQLAQN------   57
FBpp0187470      HLPFGTEQPELDVVIVGSGLSALTSAVKLLAKETTLHIRILDENSRPGGQLAQN------   57
FBpp0240881      DLPFGTQTPELDVLIVGAGLSGLASAVKILSKEQSLKMKIIDENPMPGGQLGES------   55
FBpp0238627      DLPFGNELPQIDILIVGAGLSGLTSAVKILSKEHSLNVKIIDENNKPGGQLRGK------   55
FBpp0392421      ------------------------------------------------------------   0
FBpp0171313      DLPFGNELPELDVLIVGAGLSGLTSAVKILNKVDTLNVKIIEEGSMPGGQLSSE------   55
FBpp0148092      DLPFGNDLPELDILIVGAGLAGLTSAIKILSKDSSLNMRIIDENRKPGGQLGSN------   114
MAOB-001         ------MSNKCDVVVVGGGISGMAAAK--LLHDSGLNVVVLEARDRVGGRTYTLRNQKVK   52
MAOA-001         QEKASIAGHMFDVVVIGGGISGLSAAK--LLTEYGVSVLVLEARDRVGGRTYTIRNEHVD   61
MAOA-201         ------------------------------------------------------------   0

FBpp0083984      ----STRLVDAAQQDMLALLTLIHLSPRRRRS--------------ISGSLRRCWDLDRG   97
FBpp0219486      ----STRLVDAAQQDMLALLTLIHLSPRRRRS--------------ISGRLRRCWDLDRG   97
FBpp0208059      ----STRLVDAAQQDMLALLTLIHLSPRRRRS--------------ISGRLRRCWDLDRG   97
FBpp0129819      ----STRLVDGAQQDMLALLTIIHLSPRRRRS--------------VSGRLRRCWDLDRG   97
FBpp0268476      ----GIRLVDGAQQDMLALLTLVHLSPRRRRS--------------INGRLRRCWDLDRG   97
FBpp0119313      ----GVRFIDSEQQDMLSFLNLVQLNPRQRRS--------------DSSSLKRCWDLDRG   97
FBpp0283967      ----GIRFVDEEQHDMLNFMQDMQLSLQHRQN--------------ENSQLRRCWDLDRG   99
FBpp0187470      ----GIRFVDEEQHDMLNFMHDMQLSLQHRHN--------------ENSQLRRCWDLDRG   99
FBpp0240881      ----GNRFVNSDQDQMITFLTYVQVLPHRYVPDF------------SSDSLRRCWDLDRG   99
FBpp0238627      ----GTCFVSKEQKELIAFITQLNVALFQHD--S------------HEQTLSRCWELDRG   97
FBpp0392421      ------------------------------------------------------------   0
FBpp0171313      ----GNRFVQQDQSELIAFLTQLNVAVNERKESD------------RNESLNRCWELDRG   99
FBpp0148092      ----GSRIVNKEQTELIAFLSQLNVAVRQRN--L------------NTDQLNRCWELDRG   156
MAOB-001         YVDLGGSYVGPTQNRILRLAKELGLETYKVNEVERLIHHVKGKSYPFRGPFPPVWN----   108
MAOA-001         YVDVGGAYVGPTQNRILRLSKELGIETYKVNVSERLVQYVKGKTYPFRGAFPPVWN----   117
MAOA-201         ------------------------------------------------------------   0

FBpp0083984      LTAMPAKFELGRYIDMLDLRMPKFRSKRFNLRERV--SNMEQHICHHLFFSKSRNFMLNL   155
FBpp0219486      LTALPAKFELGRYIEMLDLRMPKFRSKRFNLRERV--SNMEQHICHHLFFSKSRNFMLNL   155
FBpp0208059      LTALPAKFELGRYIEMLDLRMPKFRSKRFNLRERV--SNMEQHICHHLFFSKSRNFMLNL   155
FBpp0129819      LTALPAKFELGRYIEMLDLRMHKFRSKRFSLRERV--SNMEQHICHNLFFSKSRNFMLNL   155
FBpp0268476      LTALPAKFELGRYIEMLDLRMPKFRSKRFNLRERV--SNMEQHICHHLFFSKSRNFMLSL   155
FBpp0119313      LTSLPAKFELNRYINMLELRMSKFRSKRFNLRERV--ANMEHHICQHLYFSKSRNFMLNL   155
FBpp0283967      PTAQLAKFELCRYIQMLDLRMGRFRAKRFKLRRRM--PSMERHITQNLFFSKSRNFMRNL   157
FBpp0187470      PTAQLAKFELCRYIQMLDLRMGRFRAKRFKLRRRM--PSMERHITQNLFFSKSRNFMRNL   157
FBpp0240881      LTSLPARFELWRYINMLEIRMKKFGSKKFNLRNRG--PTMERHICNNLFFNKSRFFMFNV   157
FBpp0238627      LTSVPAKFELWRYINMLDMRMKKFSSVRYEFRERI--PTMERHIVTNLFFNLSREFMYQL   155
FBpp0392421      ------------------------------------------------------------   0
FBpp0171313      LTSTLAKFELWRYIHMLDLRMNKFDS-RYKYRTRI--PTMERHICQNLFFSLSRKFMYNL   156
FBpp0148092      LTSTPASFELWRYIEMLDLRMKKFNGLHFQFRKRV--PTMERHISTNLFFNKSRQFMFNL   214
MAOB-001         PITYLDHNNFWRTMDDMGREIPSDAPWKAPLAEEWDNMTMKELLDKLCWTESAKQLATLF   168
MAOA-001         PIAYLDYNNLWRTIDNMGKEIPTDAPWEAQHADKWDKMTMKELIDKICWTKTARRFAYLF   177
MAOA-201         ----------------MGKEIPTDAPWEAQHADKWDKMTMKELIDKICWTKTARRFAYLF   44
```

```
FBpp0083984    VQLVCGVPANEVDYDVFMSVCSSCGGLKVLIDFYFTYPTSFYEISTQMLIENILEQLQFI   215
FBpp0219486    VQLVCGVPANEVDYDVFMSVCSSCGGLKMLIDFYFTYPTSFDEISTQMLIENILEQLQFI   215
FBpp0208059    VQLVCGVPANEVDYDVFMSVCSSCGGLKMLIDFYFTYPTSFDEISTQMLIENILEQLQFI   215
FBpp0129819    VELVCGVPANEVDYDVFMSVCSSCGGLKMLIDFYFTYPSSFYEISTQRLIESILEQLQFI   215
FBpp0268476    VELVCGVPASEVDYDVFMSICSSCGGLKMLIDFYFTYPSSFYEISTEMLIESILEQLQFI   215
FBpp0119313    VEIVCGVPASEVDYDVFMSICSSCGGLNMLIDFYCNYPSSFFEISTQQLIQSILDKMHFT   215
FBpp0283967    VELICGVHAREVDYDVFMSVCSSCGGLVVLIDFYFNFPNTFLELSTQQLIGNILDKIEYI   217
FBpp0187470    VELICGVHAREVDYDVFMSVCSSCGGLVVLMDFYFNFPNTFLELSTQQLIGSILDKIEYI   217
FBpp0240881    VEIACGANPSDVDYDEFMVVCSSCGGLRMLIDLYFTFPSSLFEFSTRKLVDNIMEKIKFA   217
FBpp0238627    VHLASGMQASDINYDEFMCLCSTSGGLNVLIDLLLAMPESIMSLSCGKLLETLHEKLKYT   215
FBpp0392421    ------------------------------------MPESIMSLSCGKLLETLHEKLKYT   24
FBpp0171313    VTLASGVSAKDIKYDEFMCLCSTSGGLSVLVDLYLTMPKSLLSLSCRSLLRALLEKLRHN   216
FBpp0148092    VLLTSGLPASEINYDEFMCLCSSSGGLSILVDLFMRLPSTLMDVSCKDLLEGLLKKLKHT   274
MAOB-001       VNLCVTAETHEVSALWFLWYVKQCGGTTRIISTTNGGQERKFVGGSGQVSERIMDLLG-D   227
MAOA-001       VNINVTSEPHEVSALWFLWYVKQCGGTTRIFSVTNGGQERKFVGGSGQVSERIMDLLG-D   236
MAOA-201       VNINVTSEPHEVSALWFLWYVKQCGGTTRIFSVTNGGQERKFVGGSGQVSERIMDLLG-D   103
                                                           .   :   : . :

FBpp0083984    KIILNCRAVRLEHFKNYVQVTDDQGNKHTAQAAILAIPWNKVQKLHFEPRIPKVFLPRTT   275
FBpp0219486    KIILNCRAVRLEHFKNYVQVTDDQGNKHTAQAAILAIPWNKVQKLQFEPRIPKVFLPRTT   275
FBpp0208059    KIILNCRAVRLEHFKNYVQVTDDQGNKHTAQAAILAIPWNKVQKLQFEPRIPKVFLPRTT   275
FBpp0129819    TITLNCRAVRVEHFKNYVQVTDDQGNKHTAQAAILAIPWNKVQKLDFEPRIPKAYQPRTT   275
FBpp0268476    TITLNCRAVRLDHYKNYVQVTDDQGNRHTAQAAILAIPWNKVQKLEFVPPIPTAYQPRTP   275
FBpp0119313    GITLNCKAVKVEHFKNYVQVTDDEGYKFTAEAVILAIPWNKVQKLEFVPPIPKAFLPPPA   275
FBpp0283967    TISQNVQVVKVQHFKDYVELTDAEGKKYTAQAVILAIPWNKVQQLEFEPPIPKDFCPPP-   276
FBpp0187470    TISQNVQVVKVQHFKDYVELTDAEGEKYTAQAVILAIPWNKVQQLEFEPPIPKDFCPPP-   276
FBpp0240881    DMVMNCRATRVDHFKNYVEVSDSLGMKHTAQTVILAIPWNKVQKLEFNPPLPKEFQQTV-   276
FBpp0238627    EILNNVKAVKVEHFKNYVQVTDSHGKKHKAEAVILAIPWDKVNKLQFEPQLPRQFRIPSR   275
FBpp0392421    EILNNVKAVKVEHFKNYVQVTDSHGKKHKAEAVILAIPWDKVNKLQFEPQLPRQFRIPSR   84
FBpp0171313    EIQYDAKAIKVQHFKNYVLVTDSTGQRHIAQAVILAIPWNKVESLEFEPQLPRHYRTIGR   276
FBpp0148092    DIRSGVKAVELEHFKNYVQLTDSKGDRHIAQAVILAIPWNKVNQLRFKPQLPQQFRDFSR   334
MAOB-001       RVKLERPVIYIDQTRENVLVETLNHEMYEAKYVISAIPPTLGMKIHFNPPLPMMRNQMIT   287
MAOA-001       QVKLNHPVTHVDQSSDNIIIETLNHEHYECKYVINAIPPTLTAKIHFRPELPAERNQLIQ   296
MAOA-201       QVKLNHPVTHVDQSSDNIIIETLNHEHYECKYVINAIPPTLTAKIHFRPELPAERNQLIQ   163
                :     .  :::  : : :       . .: .* ***     .: * * :*

FBpp0083984    SRSGRKGRQVISQFQLRYGKSVWTDLGYSGNFLSSQPM-VSGH-------ECRMSSFCGY   327
FBpp0219486    SRSGQKQRQVISQFQLRYGKSVWTDLGYSGNFLSSQPL-VSGH-------ECRMSSFCGY   327
FBpp0208059    SRSGQKRRQVISQFQLRYGKSVWTDLGYSGNFLSSQPL-VSGH-------ECRMSSFCGY   327
FBpp0129819    SKSGHKRRQLISQFQLRYGKSVWTDLGYSGNFLSSQPL-VSGH-------ECRMSSFCGY   327
FBpp0268476    SKSGQRRRQLISQFRLRYGKSVWTDLGYSGNFLSAQPLLVSGH-------ECRLSSFCGY   328
FBpp0119313    PKGRQKPHRVISQFHLGYGKSFWADLGYSGNFLNTQPM-VSGH-------ESRMSTICGY   327
FBpp0283967    -KGTLKSSRLITQFSLSYKKCHWAEAGFSGNFLSSKPL-VCGH-------ECHQATYCGY   327
FBpp0187470    -KGKLNSSRLITQFSLSYKKCHWAEAGFSGNFLSSKPL-VCGH-------ECHQATYCGY   327
FBpp0240881    -KAYQKPRRVITQFYMRYPKSYWRSQGYSGHFLSSEPL-VCGR-------ECRSGIYRGY   327
FBpp0238627    R-T-KKSKSMITQYSMRYNKPVWISQGYSGQFLNIRPL-VVGY-------ALNSGEYCGY   325
FBpp0392421    R-T-KKSKSMITQYSMRYNKPVWISQGYSGQFLNIRPL-VVGY-------ALNSGEYCGY   134
FBpp0171313    G-KAKRQTGQITQFFLRYDHSYWITQGYSGLFLNVDPF-VVGY-------ESNSLHYSGY   327
FBpp0148092    I-S-RQSKFVITQFTIDYDKSYWLSKGYSGNFMNMKPL-VVGY-------EQNKNEYGGY   384
MAOB-001       ----RVPLGSVIKCIVYYKEPFWRKKDYCGTMIIDGEEAPVAYTLDDTKPEGNYAAIMGF   343
MAOA-001       ----RLPMGAVIKCMMYYKEAFWKKKDYCGCMIIEDEDAPISITLDDTKPDGSLPAIMGF   352
MAOA-201       ----RLPMGAVIKCMMYYKEAFWKKKDYCGCMIIEDEDAPISITLDDTKPDGSLPAIMGF   219
                         : :  : * .  *   .:.* ::        .                *:

FBpp0083984    VLHLPE------DQDEVLQDVVDLLAEHFG-EEMRQPLECQCFT-DEL------NVA---   370
FBpp0219486    VLHSPE------DQDEVLQDVVDLLAEHFG-EEMRQPLECQCFT-DEL------NVA---   370
FBpp0208059    VLHSPE------DQDEVLQDVVDLLAEHFG-EEMRQPLECQCFT-DEL------NVA---   370
FBpp0129819    VLHSPE------DQDEVLQDVVDLLAEHFG-EEMRRPLECQCST-DEL------NVA---   370
FBpp0268476    VLHAPE------DQDEVLQDVVDLLAEHFG-EEMRQPLECQCHT-DEL------NAA---   371
FBpp0119313    MLHSPD------EQDEALETVINLLATEFG-EDMRRPLECHCFT-DEL------NVA---   370
FBpp0283967    MVHSPE------EEGSVQQTVLDLLSSQFG-DGMRQPLQYQQST-AEL------SMV---   370
FBpp0187470    MVHSPE------EEGSVQQTVLDLLSSQFG-DGMRQPLQYQQST-AEL------SMV---   370
FBpp0240881    MMHTQD------EVDEVKQTVLDLVSEYFG-DEMQHPLEYQQST-FEL------NMA---   370
FBpp0238627    MLHTVE------EADSVRTTVLDLLAEQFG-EEMLDPLTYKQET-CEL------NTA---   368
FBpp0392421    MLHTVE------EADSVRTTVLDLLAEQFG-EEMLDPLTYKQET-CEL------NTA---   177
FBpp0171313    MLHTEE------QAGTVRDTVLDVLAEQFG-EEMLDPMNYKQLT-CEL------SST---   370
FBpp0148092    MLHDTDV-----ETATVRDTILDLLAEQFG-KEMLDPLEYKEEI-CEL------NTA---   428
MAOB-001       ILAHKARKLARLTKEERLKKLCELYAKVLGSLEALEPVHYEEKNWCEEQYSGGCYTTYFP   403
MAOA-001       ILARKADRLAKLHKEIRKKKICELYAKVLGSQEALHPVHYEEKNWCEEQYSGGCYTAYFP   412
MAOA-201       ILARKADRLAKLHKEIRKKKICELYAKVLGSQEALHPVHYEEKNWCEEQYSGGCYTAYFP   279
               ::                  : :: :  :*      *:  .     *         .
```

```
FBpp0083984      ------QHKPQTKPWHRVIWSSSSAVGTSYRSLMGGAVQSGFRAAVNAVFVVR--PQVVS   422
FBpp0219486      ------QHKPQTKPWHRVIWSSSSAVGTSYRSLMGGAVQSGFRAAVNAVFVVR--PQVVS   422
FBpp0208059      ------QHKPQTKPWHRVIWSSSSAVGTSYRSLMGGAVQSGFRAAVNAVFVVR--PQVVS   422
FBpp0129819      ------QHKPQTKPWHRVIWSSSSAVGTSYRTMMGGAVQSGFRAAVNAVFVVR--PQVVS   422
FBpp0268476      ------QHKPQTKPWHRVIWSSSSAVGTSYRSLMGGAVQSGFRAAVNAVFVVR--PQVVS   423
FBpp0119313      ------LHKPQIKPWLRIIWSSSSAVGTSYRSLMGGAVQSGVRAAVNALFVVR--PQVVS   422
FBpp0283967      ------RHKPLVTPWHRVIWSSSSTVGTNYRNLMGGAVQSGVRAAVNALYVVR--PQVVS   422
FBpp0187470      ------RHKPLVTPWHRVIWSSSSTVGTNYRNLMGGAVQSGVRAAVNALYVVR--PQVVS   422
FBpp0240881      ------LHSPQITPWHRVIWSSSAAVGTYYRNLMGGAVQSGVRAAVSALFVIR--PQVVS   422
FBpp0238627      ------IHKPQIKPWHRVIWSSSAAAATKDRNLMAGAVESGVRAAINALFVIR--PQVVC   420
FBpp0392421      ------IHKPQIKPWHRVIWSSSAAAATKDRNLMAGAVESGVRAAINALFVIR--PQVVC   229
FBpp0171313      ------LHTPQVKPWNRVIWSSSASAANSNRNLMGGAVESGLRAAINALFVIR--PQVVE   422
FBpp0148092      ------VNKPQTRPWHRIVWSSSAAVATNNRNLMGGAVESGIRAAINALFVIR--PQVVG   480
MAOB-001         PGILTQYGRVLRQPVDRI-YFAGTETATHWSGYMEGAVEAGERAAREILHAMGKIPEDEI   462
MAOA-001         PGIMTQYGRVIRQPVGRI-FFAGTETATKWSGYMEGAVEAGERAAREVLNGLGKVTEKDI   471
MAOA-201         PGIMTQYGRVIRQPVGRI-FFAGTETATKWSGYMEGAVEAGERAAREVLNGLGKVTEKDI   338
                              *  *: : :.: ...     * ***::* *** . :  :    :

FBpp0083984      WKDMLVEQPKNGQDVV------SAGRIRGLLSRLNLYNVTFYSFFVIGLIWLLNLGYARS   476
FBpp0219486      WKDMLVDRPKNGQDVV------SAGRIRGLLSRLNLYNVTFYSFFVIGLIWLLNLGYARS   476
FBpp0208059      WKDMLVDRPKNGQDIV------SAGRIRGLLSRLNLYNVTFYSFFVIGLIWLLNLGYARS   476
FBpp0129819      WKDMLVERPKSAQDVV------SSGRIRGLLSRLNLYNVTFYSFFVLGLIWLLNLGYARA   476
FBpp0268476      WKDMLVERPKLAQDVV------ASGRIRGLLSRLNLYNVTFYSFFVLGLIWLLNLGYARA   477
FBpp0119313      WKDMLDDREKGSGERA------STGILTGLFSRLNLYNVTFYSFFVLGLIWLLNLGYSRA   476
FBpp0283967      WKDLLEVRETNPYERC-----SPPSRLSGLLSRLNLYNVTFYSFFFLGLIWLLKIGYCHS   477
FBpp0187470      WKDLLEVRETNPYERG-----SPPSRLSGLLSRLNLYNVTFYSFFFLGLIWLLKIGYCHS   477
FBpp0240881      WKDMMDVDEKDQYAGV------NVGRVRGLLSRLNLYNVTFYSVFVLGIIFILNIGYNRS   476
FBpp0238627      WRDLQDVQDKNRYVGP------SPGYFAGLLSRLNLYNITFYGVFVIGLICLLNFGYGAI   474
FBpp0392421      WRDLQDVQDKNRYVGP------SPGYFAGLLSRLNLYNITFYGVFVIGLICLLNFGYGAI   283
FBpp0171313      WQDLSDVHERNRYAGV------STSYISGLLSRLNLYNIAYYSVFVVGLICVLSYSYNQS   476
FBpp0148092      WRDLLDVQQKDRYAGI------SPGYFASLLSRLNLYNITFYGIFVLGLICLLNFGYD--   532
MAOB-001         WQSEPESVDVPAQPITTTFLERHLPSVPGLLRLIGLTTI--FSATALGFLA---HKRGLL   517
MAOA-001         WVQEPESKDVPAVEITHTFWERNLPSVSGLLKIIGFSTS--V--TALGFVL---YKYKLL   524
MAOA-201         WVQEPESKDVPAVEITHTFWERNLPSVSGLLKIIGFSTS--V--TALGFVL---YKYKLL   391
                 * .                       . .*:  :.: .        :*::

FBpp0083984      I---   477
FBpp0219486      I---   477
FBpp0208059      I---   477
FBpp0129819      F---   477
FBpp0268476      F---   478
FBpp0119313      T---   477
FBpp0283967      NLSS   481
FBpp0187470      NLSS   481
FBpp0240881      VS--   478
FBpp0238627      G---   475
FBpp0392421      G---   284
FBpp0171313      V---   477
FBpp0148092      ----   532
MAOB-001         VRV-   520
MAOA-001         PRS-   527
MAOA-201         PRS-   394
```

Figure S3. Protein sequence alignment of Shps and its homologous proteins of 12 species of *Drosophila* (predicted amino acid sequences were obtained from FlyBase, Gramates *et al.* 2017 FlyBase at 25: Looking to the future. Nucleic Acids Res. **45:** D663–D671) and human monoamine oxidases (predicted amino acid sequences were obtained from Ensembl, Yates *et al.* 2016 Nucleic acids research **44:** D710–D716), generated by Clustal Omega (Sievers *et al.* 2011 Fast, scalable generation of high-quality protein multiple sequence alignments using Clustal Omega. Mol. Sys. Biol. **7:** 539). To obtain colored alignment, the online web service version run by the European Bioinformatics Institute (EMBL-EBI) was used (http://www.ebi.ac.uk/Tools/msa/clustalo/). FBpp0083984, *D. melanogaster* CG13611-PA; FBpp0219486, *D. simulans* GD21084-PA; FBpp0208059, *D. sechellia* GM26582-PA; FBpp0129819, *D. erecta* GG11273-PA; FBpp0268476, *D. yakuba* GE23466-PA; FBpp0119313, *D. ananassae* GF16121-PA; FBpp0283967, *D. pseudoobscura* GA12404-PA; FBpp0187470, *D. persimilis* GL23363-PA; FBpp0240881, *D. willistoni* GK11738-PA; FBpp0238627, *D. virilis* GJ24210-PA; FBpp0392421, *D. virilis* GJ24210-PB; FBpp0171313, *D. mojavensis* GI22096-PA; FBpp0148092, *D. grimshawi* GH14186-PA; MAOB-001, *H. sapiens* Human monoamine oxidase B; MAOA-001, *H. sapiens* Human monoamine oxidase A; MAOA-201, *H. sapiens* Human monoamine oxidase A. Human monoamine oxidase A has two splice variants. The residues are colored according to their physicochemical properties: red, Small (small+ hydrophobic (incl.aromatic -Y)): AVFPMILW; blue, Acidic: DE; magenta, Basic - H: RK; green, Hydroxyl + sulfhydryl + amine + G: STYHCNGQ. * (asterisk), a single, fully conserved residue, : (colon), conservation between groups of strongly similar properties, roughly equivalent to scoring > 0.5 in the Gonnet PAM 250 matrix, . (period) indicates conservation between groups of weakly similar properties, roughly equivalent to scoring ≤ 0.5 and > 0 in the Gonnet PAM 250 matrix.

Table S1: Multiple comparisons by Dunn's methods after Kruskal-Wallis test for sperm motion parameters

A. Beat frequency: Kruskal-Wallis test H = 12.383, df = 3, P = 0.00618

		Seminal vesicle		Uterus
		shps/TM3	*shps*	*shps/TM3*
Seminal vesicle	*shps*	0.2120		
Uterus	*shps/TM3*	0.0099	0.1724	
	shps	0.1827	0.4986	0.2383

B. Beat speed: Kruskal-Wallis test H = 9.9209, df = 3, P = 0.01925

		Seminal vesicle		Uterus
		shps/TM3	*shps*	*shps/TM3*
Seminal vesicle	*shps*	0.2120		
Uterus	*shps/TM3*	0.0099	0.1724	
	shps	0.1827	0.9687	0.2383

C. Intensity index: Kruskal-Wallis test H = 25.888, df = 3, P < 0.0001

		Seminal vesicle		Uterus
		shps/TM3	*shps*	*shps/TM3*
Seminal vesicle	*shps*	0.0027		
Uterus	*shps/TM3*	0.0005	0.1220	
	shps	0.0071	0.1310	0.6430

Probabilites are adjusted with the Benjamini-Hochberg method (R Core Team, 2017 R: A Language and Environment for Statistical Computing. R Foundation for Statistical Computing, Vienna, Austria).

Table S2: Strains used in this study

Strain	Genotype	Stock availability
C(1)RM, y v f/C(1;Y)6; shps/TM3, Sb Ser	*C(1)RM, y*^{1} *v*^{1} *f*^{1}*/C(1;Y)6; shps*^{1}*/TM3, Sb*^{1} *Ser*^{1}	Upon request
Canton-S	(Wild type)	Kyoto DGRC# 105666
crb^{j1B5}*/TM3*	*y*^{1} *w*^{1118}*; P{w*$^{+mC}$*=lacW}crb*^{j1B5}*/TM3, Sb*^{1}	Bloomington Stock# 10331
Df(3R)crb-F89-4	*Df(3R)crb-F89-4, st*^{1} *e*^{1}*/TM3, Ser*^{1}	(Kyoto DGRC# 107742) [a]
Df(3R)crb87-4	*Df(3R)crb87-4, st*^{1} *e*^{1}*/TM3, Ser*^{1}	Bloomington Stock# 2362
Df(3R)crb87-5	*Df(3R)crb87-5, st*^{1} *e*^{1}*/TM3, Ser*^{1}	Bloomington Stock# 2363
Df(3R)Exel6198	*w*^{1118}*; Df(3R)Exel6198, P{w*$^{+mC}$*=XP-U}Exel6198/TM6B, Tb*^{1}	Bloomington Stock# 7677
Df(3R)Exel6199	*w*^{1118}*; Df(3R)Exel6199, P{w*$^{+mC}$*=XP-U}Exel6199/TM6B, Tb*^{1}	Bloomington Stock# 7678
Df(3R)Exel8178	*w*^{1118}*; Df(3R)Exel8178/TM6B, Tb*$^{+}$	Bloomington Stock# 7993
Df(3R)P3-4-7	*w***; Df(3R)P3-4-7, P{w*$^{+mC}$*=lacW}degenerated/TM6B, Tb*^{1} *Sb*^{1}	Kyoto DGRC# 118172
GFP-myosin VI	*w***; P{w*$^{+mC}$*=Hsp83-jar.GFP}; Df(3R)crb87-5, st*^{1} *e*^{1}*/TM3, Sb*^{1}	Miller (Noguchi *et al.* 2006) [b]
In(3R)Ubx^{7LL}*ats*^{R}	*In(3R)Ubx*^{7LL}*ats*^{R}*, asp*^{1} *ats*^{1} *p*^{p}*/TM6B, Tb*^{1} *ca*^{1}*; y*^{1}*/Dp(1;Y)y*$^{+}$	Bloomington Stock# 1972
jar^{1}*/TM3*	*ry*^{506} *P{ry*$^{+t7.2}$*=PZ}jar*^{1}*/TM3, ry*^{RK} *Sb*^{1} *Ser*^{1}	Bloomington Stock# 11793
Oregon-R	(Wild type)	Upon request
PBac{CG13611^{+}*}VK00037 (S2)*	*w***; PBac{w*$^{+mC}$*=UAS-CG13611}VK00037-S2*	Kyoto DGRC# 118173
PBac{CG13611^{+}*}VK00037 (S5)*	*w***; PBac{w*$^{+mC}$*=UAS-CG13611}VK00037-S5*	Kyoto DGRC# 118174
P{protamineB-eGFP}	*w***; P{w*$^{+mC}$*=protamineB-eGFP}1/CyO*	Kyoto DGRC# 109173
ru h th st cu sr e^{s} *ca/TM3, Sb Ser*	*ru*^{1} *h*^{1} *th*^{1} *st*^{1} *cu*^{1} *sr*^{1} *e*^{s} *ca*^{1}*/TM3, Sb*^{1} *Ser*^{1}	Kyoto DGRC# 101738
shps/TM3	*shps*^{1}*/TM3, Sb*^{1} *Ser*^{1}	Kyoto DGRC# 118171
w**; Dr/TMS, P{Δ2-3}99B*	*w***; Dr*^{1}*/TMS, P{ry*$^{+t7.2}$*=Δ2-3}99B*	Kyoto DGRC# 106416
y M{vas-int.Dm}ZH-2A w**; PBac{y*$^{+}$*-attP-3B}VK00037*	*y*^{1} *M{vas-int.Dm}ZH-2A w***; PBac{y*$^{+}$*-attP-3B}VK00037*	Bloomington Stock# 24872

Bloomington: Bloomington Drosophila Stock Center, Indiana University (http://flystocks.bio.indiana.edu/)

Kyoto: KYOTO Stock Center (DGRC), Kyoto Institute of Technology (https://kyotofly.kit.jp/stocks/)

Miller: Dr. K. Miller at Washington University

Upon request: Available upon request to the authors

[a] The strain used in this study was obtained from Bloomington Drosophila Stock Center and its stock# was 4432.
However, its genotype is different from the present stock at Bloomington Drosophila Stock Center (#4432 has a different balancer chromosome).
The subline of the previous version of the stock (used in this study) is available at KYOTO Stock Center (DGRC# 107742).

[b] Noguchi, T., M. Lenartowska, and K. G. Miller, 2006 Myosin VI stabilizes an actin network during *Drosophila* spermatid individualization. Mol. Biol. Cell **17:** 2559–2571.

SUPPLEMENTAL FILE LEGENDS

Supplemental File S3. Sperm motion video at 10× slowed down (30 frames/s), captured by a high-speed camera (300 frames/s). Four movies are combined into a single Audio Video Interleave (AVI) file. (Upper Left) Sperm dissected from seminal vesicle of *shps/TM3* male. (Lower Left) Sperm dissected from uterus of female that copulated with *shps/TM3* male. (Upper Right) Sperm dissected from seminal vesicle of *shps* male. (Lower Right) Sperm dissected from uterus of female that copulated with *shps* male.
